# Advances in fatty acids nutrition in dairy cows: from gut to cells and effects on performance

**DOI:** 10.1186/s40104-020-00512-8

**Published:** 2020-11-16

**Authors:** Massimo Bionaz, Einar Vargas-Bello-Pérez, Sebastiano Busato

**Affiliations:** 1 Department of Animal and Rangeland Sciences, Oregon State University, Corvallis, OR 97331 USA; 2 Department of Veterinary and Animal Sciences, Faculty of Health and Medical Sciences, University of Copenhagen, Grønnegårdsvej 3, DK-1870 Frederiksberg C, Denmark

**Keywords:** Absorption, Dairy cow, Dietary fatty acids, Intestine, Liver, Metabolism, Performance, Transcription factors, Transport

## Abstract

High producing dairy cows generally receive in the diet up to 5–6% of fat. This is a relatively low amount of fat in the diet compared to diets in monogastrics; however, dietary fat is important for dairy cows as demonstrated by the benefits of supplementing cows with various fatty acids (FA). Several FA are highly bioactive, especially by affecting the transcriptome; thus, they have nutrigenomic effects. In the present review, we provide an up-to-date understanding of the utilization of FA by dairy cows including the main processes affecting FA in the rumen, molecular aspects of the absorption of FA by the gut, synthesis, secretion, and utilization of chylomicrons; uptake and metabolism of FA by peripheral tissues, with a main emphasis on the liver, and main transcription factors regulated by FA. Most of the advances in FA utilization by rumen microorganisms and intestinal absorption of FA in dairy cows were made before the end of the last century with little information generated afterwards. However, large advances on the molecular aspects of intestinal absorption and cellular uptake of FA were made on monogastric species in the last 20 years. We provide a model of FA utilization in dairy cows by using information generated in monogastrics and enriching it with data produced in dairy cows. We also reviewed the latest studies on the effects of dietary FA on milk yield, milk fatty acid composition, reproduction, and health in dairy cows. The reviewed data revealed a complex picture with the FA being active in each step of the way, starting from influencing rumen microbiota, regulating intestinal absorption, and affecting cellular uptake and utilization by peripheral tissues, making prediction on *in vivo* nutrigenomic effects of FA challenging.

## Introduction

Important advances in the understanding of overall lipid digestion, absorption, and metabolism in dairy cows has been made between 1950 to 1990; afterwards, due to technological advances, a larger emphasis was placed on determining the molecular aspects of those processed. Furthermore, before the 1990’s, the effect fatty acids (FA) on transcription of genes was virtually unknown. Discovery of transcription factors (TF) that sense the presence of lipids, especially FA, and the advances in the understanding of biological effects of FA in many pathways and functions in cells, have provided a great window into the biological roles of FA. Borrowing methods and knowledge obtained from monogastric species, especially model organisms, advances were made on our knowledge on the molecular processes related to the metabolism and biological functions of lipid molecules in dairy cows.

Different from monogastric species, ruminants generally receive a low amount of lipids into the diet. It is common practice in nutrition of dairy cows to provide no more than 6% of lipids (> 90% FA) in the diet, with around 3% coming from the forages and grains, and the rest added as supplemental fat. Different than monogastrics where dietary FA arrive to the intestine with no modifications, the presence of the rumen with the high microbial activity, substantially modify FA, especially unsaturated FA (UFA); thus, the need of supplementing dairy cows rumen-protected FA. A recent review about the historical prospect and importance of feeding fat to dairy cows, especially rumen-protected FA, is available elsewhere [1]. The supplementation of fat has positive effects on the performance of dairy cows, as observed with UFA (if rumen protected) [2] and saturated FA (SFA), especially C16:0 and C18:0 [3]. The observed positive effects of supplementing rumen-protected fat to dairy cows goes beyond the increased level of energy in the diet; however, this additional effect remains largely unexplained and can be considered a “black box” in molecular nutrition and physiology of dairy cows.

Nutrigenomics is a relatively new branch of science in dairy cows with the underline hypothesis that feedstuffs contain compounds that directly affect transcription of genes via binding of specific TF [4]. In this contest, few are the TF that can be modulated by FA [4]. Among those, the peroxisome-proliferator-activate receptors (PPAR) and Sterol regulatory element-binding protein (SREBP) have been studied extensively in ruminants, as previously reviewed [4, 5]. Besides TF also free fatty acid receptors are emerging as important player in the transcriptomic response to FA. Nutrigenomics is thus of importance in dairy cows when considering the observed beneficial effects of dietary FA. The possibility of finely modulating specific TF by dietary FA hold a great promise in improving animal health and performance, but also improving milk quality [4, 6, 7].

The application of nutrigenomic approaches by supplementing dairy cows with FA requires understanding at molecular level of all the processes of FA in dairy cows once ingested, from the digestion, absorption, and transport in circulation to cellular uptake and effects in peripheral tissues. Here we attempt to provide an up-to-date review of all those aspects with the intent to contribute to shine some light into the nutrigenomic use of dietary FA in dairy cows and their effects on the biology of dairy cows. Furthermore, we cover the latest effects of supplementing FA on performance and health of dairy cows.

## Fate of dietary lipids in the rumen

Excellent comprehensive reviews about the digestion, absorption and transport of lipids in ruminant animals were provided more than 20 years ago by Noble [8], Jenkins [9], and Harfoot and Hazlewood [10]. More recent reviews have covered lipid metabolism in the rumen [11, 12] and very little advances have been made since the publications of those reviews.

From the point of view of nutrition of ruminants, it is important to highlight that forages contain around 2% lipids, mostly in the form of galactosyldiglycerides and phospholipids, with minor amount of tri- and di-glycerides. The most abundant FA present in forages are C18:3 (60–70%) and C18:2 (~ 20%). Triglycerides (TAG) are instead the most abundant form of lipids when seed-derived products are fed to dairy cows [13]. Lipids in the rumen, when released via mastication and microbiota activity, undergo two major processes: lipolysis and biohydrogenation [12].

### Lipolysis

The lipolysis of esterified FA in the rumen is performed by lipases released by rumen bacteria, mainly *Anaerovibrio lipolytica* [10, 12, 13] that break down mostly TAG, and *Butyrivibrio* spp. responsible for hydrolysis of phospho- and galacto-lipids, but also by lipases present in fresh plant materials. Those lipases remain active for up to 5 h in the rumen [13]. A characterization of the lipases present in bacteria using a combination of a genomic approach and expression of recombinant proteins identified three lipases in *Anaerovibrio lipolyticus* with high activity toward MCFA, particularly C8:0, C10:0, and C12:0 [14]. Interestingly, the relative abundance of those bacteria increases early post-partum when the diet is enriched with large amount of fat, such as cottonseed and rumen inert fat [15]. In addition, galactosidases and phospholipases from the plants participate to the release of FA and the use of FA to produce energy by rumen microbes is minimal, manly by protozoa [9].

### Biohydrogenation

The FA released by lipolysis are rapidly and almost completely hydrogenated by the action of bacteria isomerases followed by the activity of reductases [9]. The attachment of FA to food particles in the rumen increases biohydrogenation, while bacteria adherent to particles tend to accumulate more UFA and protect them from biohydrogenation [10]. More than 60% of free FA are adsorbed onto the surface of the feed particles present in the rumen [9]. Protozoa in the rumen play a role in accumulation of dietary FA as lipid droplets in their cells. Protozoa appears also to accumulate and provide between 30% and 40% of ruminal outflow of biohydrogenation intermediates, such as conjugated linoleic acids (CLA) and vaccenic acid [13].

As reviewed by others [10–12], there are several types of bacteria that can induce biohydrogenation of UFA in the rumen. The biohydrogenation of UFA requires the presence of a free carboxyl group. Due to the pathway involved in biohydrogenation, the main CLA escaping the rumen are *cis*-9, *trans*-11 and *trans*-9, *trans*-11 while *trans*-11 is the most abundant from C18:1, but many more positional and geometric isomers of CLA outflow the rumen and are present in milk of dairy cows [11, 16]. A very recent study demonstrated that PUFA esterified to more complex lipids, such as phospholipids and cholesterol esters proportionally abundant in forages, are less prone to biohydrogenation compared to PUFA esterified to TAG, abundant in seeds and derived oil [17]. Findings from that study can have important implication in feeding dairy cows to increase enrichment of PUFA in milk.

The large biohydrogenation of UFA in the rumen is the major impediment to enrich cattle products with UFA, especially poly-unsaturated FA (PUFA) [13]. Several techniques have been developed to protect FA from rumen biohydrogenation, including encapsulating FA in a matrix of protein treated with formaldehyde, production of calcium soap, heat treatment, or conversion of FA to fatty acyl amide [1, 9]. The protection of FA from rumen microbiota is however only partial [18].

### Lipids leaving the rumen

Around 20% of lipids leaving the rumen are of microbial origin (or an estimated 15 g/kg of organic matter in animals not receiving lipid supplement [9]), mainly bacteria and protozoa. Of those, around 70% are neutral lipids produced by microorganisms partly via *de novo* synthesis using acetate and glucose to produce even-chain FA, mostly C18:0 and C16:0 at a ratio of 2:1, and propionate for the synthesis of odd-chain FA, such as C13:0, C15:0, and C17:0 [9] with minor amounts of rumen biohydrogenation intermediates [10].

### Volatile fatty acids and their contribution to lipid metabolism in dairy cows

Forty years ago it was very well-established that rumen microbes produce volatile fatty acids (VFA) that are chiefly absorbed by the epithelium of the rumen and of the omasum (> 70% of all the produced VFA) [8]. Among VFA, acetate (60–70% of all VFA) and the butyrate-derived β-hydroxybutyrate (BHBA) are the main precursors for *de novo* synthesis of FA in peripheral tissues, with a minor role of propionate for the synthesis of odd-chain FA [19]. Acetate has been estimated to contribute between 70% and 80% of acetyl-groups for lipogenesis in adipose tissue, 15–30% in intramuscular depots [20], and it is the major precursor for *de novo* FA synthesis in mammary gland [21, 22]. The rumen is the major locus for the synthesis of circulating BHBA both from the absorbed butyric acid (10–15% of all VFA; approx. half of absorbed butyric acid is converted to BHBA) and medium-chain FA (MCFA) [9]. The MCFA and long-chain FA (LCFA) are absorbed at negligible amount (estimated to be < 4%) by the pre-stomachs and abomasum where they are used for production of BHBA by the rumen and omasum epithelium, especially the MCFA. More recently, it was discovered that specific proton-linked monocarboxylate transporters (MCT or SLC16A) are responsible for the transport of short-chain fatty acids (SCFA), with the MCT1, MCT2, and MCT4 likely playing a major role in forestomach absorption of SCFA [23, 24]. However, according to Graham and collaborators [25], the absorption of SCFA by the rumen epithelium is mainly driven by a non-saturable Na^+^/H^+^ antiporter (SLC9A) at the apical part of the epithelial cells. The Na^+^/H^+^ exchangers allow the flow of SCFA from the lumen to the cytosol by exchanging Na with protonated SCFA. The MCT are instead responsible for the translocation of SCFA (or their metabolites, mainly BHBA) from the epithelial cells to the capillaries [24]. Despite the need of saturable transporters, the absorption of SCFA is highly associated with the magnitude of rumen epithelial blood flow. Additional, SCFA can be transported by the sodium-coupled monocarboxylate-transporter 1 (SMCT1/SLC5A8) [26], which appears to transport butyrate and it is highly expressed in the rumen epithelium, but its expression is reduced *in vivo* by the increase concentration of butyrate [27].

### Effects of fatty acids on microbiota of the rumen

Around 20% of *de novo* synthesized FA by bacteria are monounsaturated FA. The *de novo* FA synthesis of bacteria as well as overall bacterial fermentation are however reduced when FA are increased in the diet, especially by fat supplementation. Jenkins reported that 10% of fat added into the diet can reduce the fermentation by > 50%, especially if UFA are supplemented [9]. The inhibition of fermentation appears to be due to a coating effect of FA to feed particles but also to a toxic effect of FA by disrupting bacteria membranes [9].

The negative effects of UFA on rumen microbiota have been known for more than five decades. Use of advanced microbiota analysis revealed a strong effect of dietary FA on microbiota composition, as recently reviewed [13, 28]. PUFA appears to be more toxic to bacteria compared to less unsaturated counterparts [12, 13]. The effect is quite substantial on the overall microbiota composition and activity, as recently demonstrated in goats where supplementation of PUFA-rich linseed substantially reduced the microbiota biodiversity [29]. Considering the negative effect of UFA on bacteria, it has been proposed that biohydrogenation is the main process whereby bacteria reduce the toxicity of those types of FA [13, 28].

Saturated FA can be somewhat toxic to rumen bacteria. Palmitic and stearic acid have been shown to be toxic but only to *Prevotella ruminicola* (propionate producer) and some strains of *Butyrivibrio fibrisolvens* (acetate and butyrate producers) when added to purified bacterial culture, but the toxicity is less than oleic acid [28]. Among SFA, lauric acid (C12:0) appears to be the most bioactive on rumen microbiota. Early studies using supplementation of C12:0 observed an inhibitory effect on protozoa [28], which should improve ruminal efficiency (especially N utilization) and milk production. The same finding was confirmed by more recent studies [30, 31]. The latter studies however concluded that, despite there was a reduction of protozoa, addition of up to 540 g/d of lauric acid was not enough to improve ruminal efficiency. Lauric acid has antimicrobial properties when fed to broilers or pigs [32, 33]. The effect of lauric acid on rumen bacteria is less known. Recent studies revealed an inhibitory effect of C12:0 and myristic acid (C14:0) on rumen methanogenesis [34–36]. The MCFA have also important effects in reducing rumen biohydrogenation, especially capric acid (C10:0) [13].

## Fate of FA in the intestine

There is a fundamental difference in the digestive system between monogastrics and ruminants. In monogastrics, the duodenum receives intermittent large boluses containing mostly TAG, and the neutralization of each bolus is very rapid. In ruminants the amount of digesta coming from the abomasum is homogenous and is constituted of fine particles, but the quantity of continuous acid material is massive (200–1200 mL/h) [8]. Thus, the digesta in the duodenum of ruminant is not fully neutralized, but remains acidic until reaching the end of the jejunum (pH of 3 in duodenum and upper jejunum and 6 in distal jejunum) [8, 37]. In ruminants, the large majority of lipids flowing through the small intestine are free FA that form micelles or are attached to food particulates [8, 37]. In ruminants the pancreatic lipase is less important than the bile [8], considering that the latter helps dissociate the micelles of FA and also helps dissociate the FA intimately associated with dietary particulates. In normal dietary conditions, i.e., with not added esterified fatty acids, the amount of TAG coming from the abomasum is very low in ruminants and, thus, the importance of lipases is minimal [8], which is contrary to monogastrics [38].

### Absorption of FA by enterocytes

In monogastric the concentration of free FA in solution in the intestinal lumen is relatively high, in the order of μmol/L, mostly as consequence of the high activity of the lipases [39]. Due to the low concentration of lipids in the diet of ruminants, the low activity of lipases, and the large amount of bile forming micelles, it is likely that the concentration of free FA in solution of the intestinal lumen is lower compared to monogastric animals.

Fatty acids reach the outer membrane of the enterocytes mainly incorporated in micelles. The latter allow overcoming the unstirred water layer close to the surface of the enterocytes.

The low pH in the small intestine has important implications for the absorption of FA. Low pH can negatively affect the aggregation of micelles [40] decreasing their solubility and digestibility but also increases the dissociation of micelles containing FA and the hydrogenation of FA, increasing their absorption [41]. Low pH inhibits the activity of pancreatic lipase in the upper part of the intestine, so the release of FA from TAG by this enzyme happens only after reaching a higher pH in the middle of the jejunum [37]. In ruminants, taurine is the predominant component of the bile, and at low pH, it is partially ionized favoring solubilization and formation of micelles [8, 38, 42].

The combination of a large amount of bile and the lower pH likely induces a high release of free FA from the micelles into the unstirred water layer close to the enterocytes. This might explains the high FA absorption efficiency in ruminants compared to monogastrics, particularly for SFA [8]. The efficiency of absorption of FA in ruminant goes from 75% for SFA to 80–90% for PUFA [43, 44]. The efficiency of FA absorption is negatively affected by the level of C18:0 flowing into the duodenum [44] and by the level of dietary fat, with a reduction from 95% to 78% of FA absorption when dietary lipids increase from 1% to 8% [37]. In monogastric animals the absorption of FA in the jejunum is lower than ruminants, especially for the SFA (17% for C18:0, 50% for C16:0, C18:1 and C18:2, and 65% for C14:0), as quantified in rats [45]. Higher values were observed in humans, where > 95% absorption was reported for FA present in fish oil besides eicosapentaenoic and docosahexaenoic acids (DHA) that had an absorption of < 70% [46]. As for other species, in human SFA are absorbed with lower proportion compared to UFA [47]. Thus, despite the lower amount of FA in the diet of dairy cows, the supplementation of FA, if adequately rumen protected, can be highly effective.

A physical barrier for the FA to be absorbed by enterocytes is the mucus covering the enterocytes [42, 48]. The main constituent of mucus are the various mucins. In the intestine mucin 1 plays a major role and appears to be essential for absorption of cholesterol but not FA in mice [49]. In dairy cows mucin 1 and mucin 20 are expressed through the whole GI tract, but their expression is higher in the small intestine [50]. There are no data on the role of mucin on lipid absorption on bovine.

Once the FA reach the outer membrane of the enterocytes, it is absorbed by both diffusion and active transport (see more details on “Cellular fate of fatty acids” section). The relative importance of each of those transport systems in the intestine absorption of FA is still debated. There are authors indicating diffusion being quantitatively more important [39] while others indicate the active transport via specific translocases proteins playing a major role by docking the luminal FA to the membrane of enterocytes that are then passively diffused and are re-docked on the inner side of the plasma membrane [42]. In monogastrics, the argument for the passive diffusion during absorption of intestinal FA is based on the large concentration in solution of free FA in the intestinal lumen. Due to the low amount of lipids in the diet and the high amount of SFA, which have lower solubility compared to UFA [8], the concentration of free FA in the intestinal lumen is very likely low in ruminants, indicating the active transport being more important.

### Molecules involved in intestinal FA absorption

**Fig. 1:**
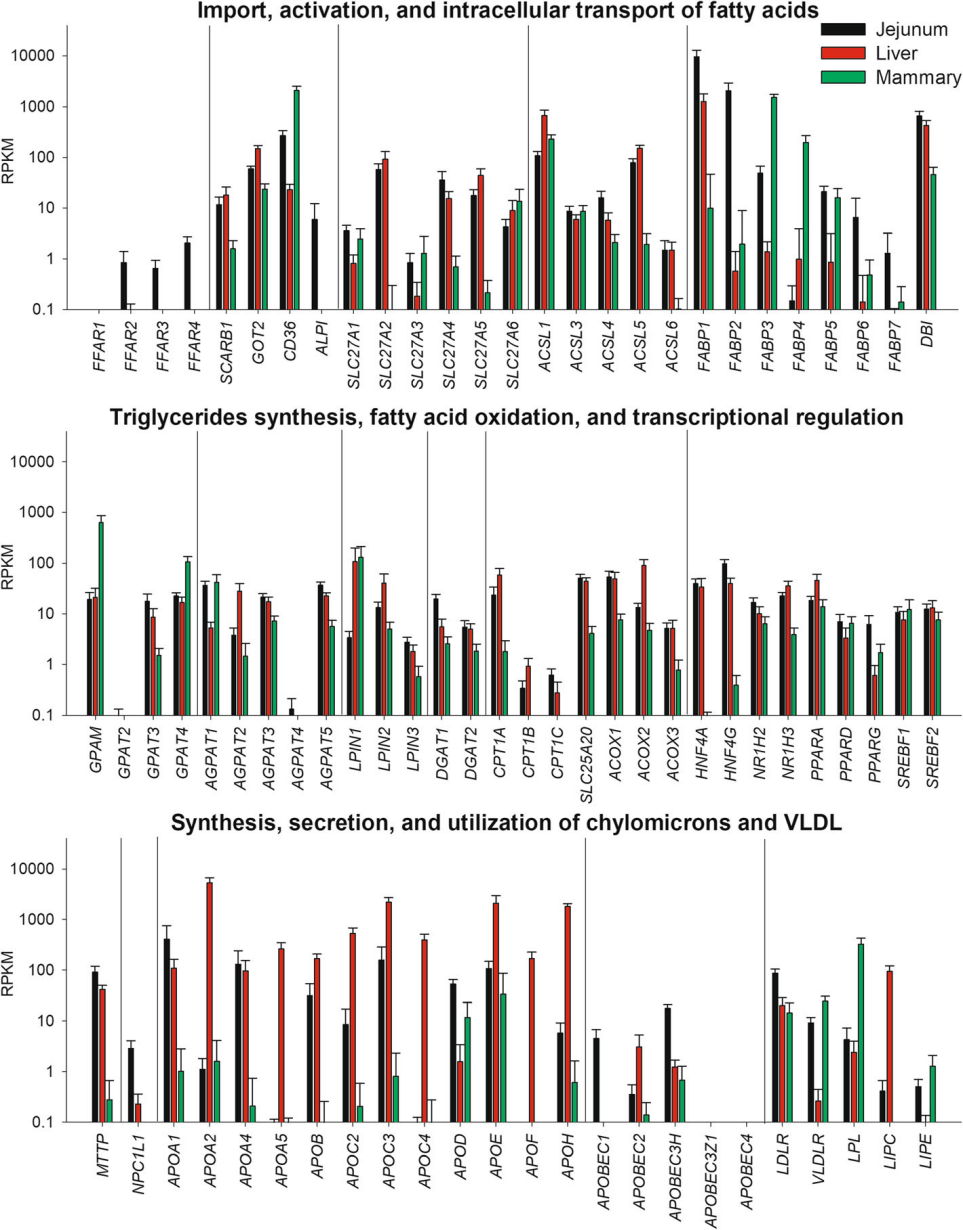
Transcript abundance of various gene isoforms involved in fatty acid absorption in jejunum, liver, and mammary tissue from Chinese Holstein lactating cows [54]. Data were downloaded from Gene Expression Omnibus, dataset GSE78524. Data are mean ± SD of reads per kilo base per million mapped reads (RPKM) of all 18 cows

**Fig. 2:**
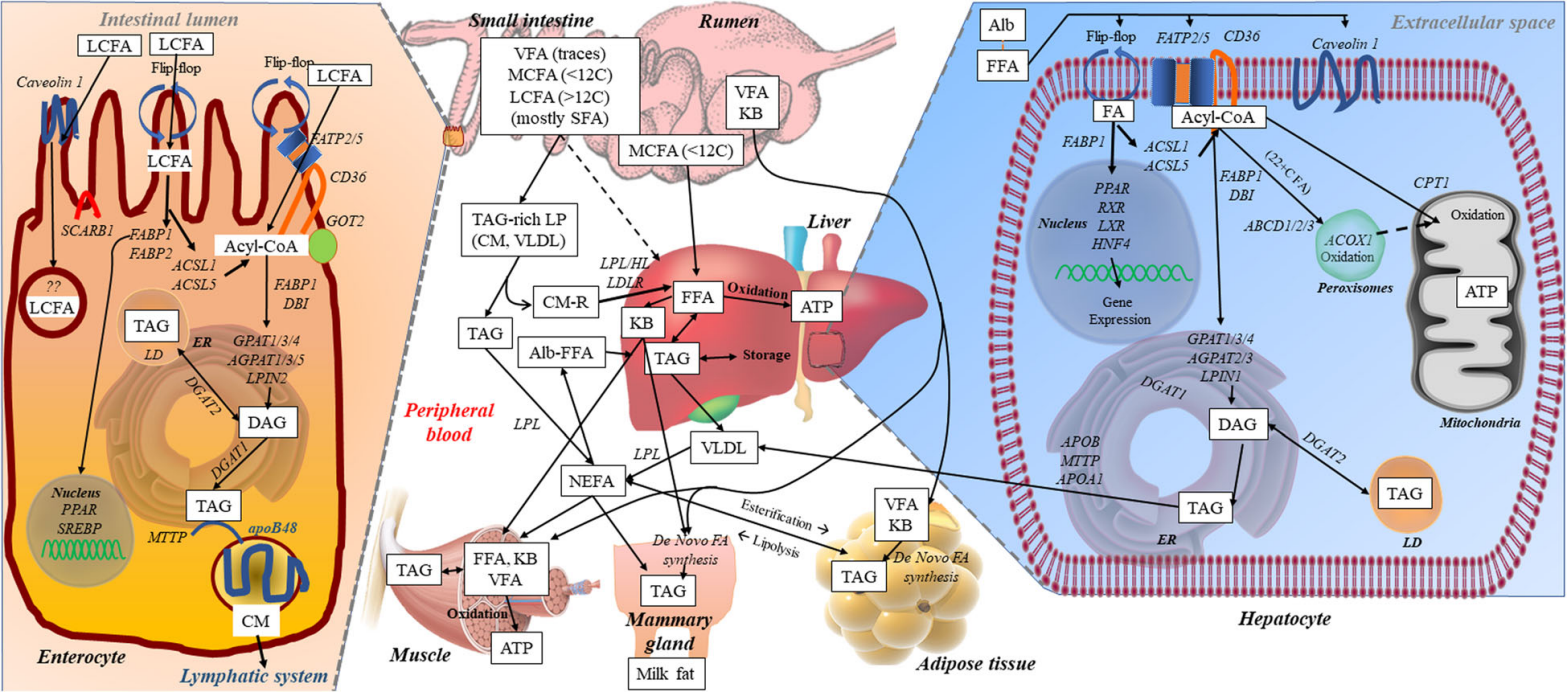
Model summarizing in dairy cows the absorption of fatty acids by enterocytes and their utilization by peripheral tissues (especially liver) with indicated enzymes and other proteins/complexes involved. As discussed in detail in the review, the model is mostly based on data obtain from monogastric species together with available data in ruminants, including the transcription abundance of the various genes presented in Fig. 1. Abbreviations: Alb, albumin; CM, chylomicron; CM-R, chylomicron remnants; DAG, diacylglycerol; ER, endoplasmic reticulum; FA, fatty acids; FFA, free fatty acids; KB, ketone bodies; LCFA, long chain fatty acids; LD, lipid droplets; LP, lipoprotein(s); LPL, lipoprotein lipase; MCFA, medium chain fatty acids; NEFA, non-esterified fatty acids; TAG, triacylglycerol; TAG-rich LP, TAG-rich lipoproteins; VFA, volatile fatty acids; VLDL, very low density lipoproteins

There are no studies in ruminants about molecular absorption of FA from the intestine and most of the information available are from studies conducted in monogastrics, chiefly using murine model, with some studies also carried out in pigs (e.g., [51, 52]). Several proteins drive the active absorption of FA in the enterocytes: fatty acid translocase CD36, scavenger receptor B1 (SR-B1), membrane-associated fatty acid binding protein (FABPpm), and the various fatty acid transport proteins (FATP) [39, 42, 53]. Transcript abundance of those molecules in jejunum, liver, and mammary tissue of lactating cows is available in Fig. 1. A model summarizing the various steps in FA absorption by the enterocytes is available in Fig. 2.

Intestinal cell line models for bovine have been established from the duodenum [55] or other intestinal sections [56] but have not been used to study absorption of FA. Furthermore, FA are known to be bioactive in bovine cells by affecting the transcriptome [4]. However, studies on the transcriptomic effect of FA on intestinal epithelial cells of ruminants are scant. The only data available are from a study performed on goat intestinal jejunum cells where SCFA induced the transcription of genes related to immune response and SCFA absorption [57].

#### Main mechanisms of transport of fatty acids across cytoplasmic membrane

From a biochemical perspective, the plasma membrane is composed of a two leaflets of lipids, interleaved by structural, membrane-embedded proteins, and peripheral proteins [58]. A large degree of dynamism is present between its main structural components; individual phospholipids in the plasma membrane rotate very rapidly along their axis, and can diffuse laterally at a considerable rate, switching position with a neighboring lipid within ~ 100 nanoseconds [59]. In stark contrast with this, transbilateral diffusion (from one leaflet to the other) occurs quite slowly and is dependent on the type of lipid in consideration, particularly the polarity of the headgroup. Lipids with a simple hydroxyl headgroup, such as ceramide, cholesterol, and diacylglycerol, have a high rate of spontaneous transbilayer diffusion [60]; in general, the movement of a lipid’s polar headgroup through the hydrophobic inner space of the plasma membrane is highly energetically unfavorable, and as such, benefits from being facilitated by membrane proteins.

Cross-membrane permeation is achieved in a three-step process [61]: 1) FA move from the aqueous phase to the surface of the plasma membrane; 2) FA “flip-flop”, moving from one half of the bilayer to the other; 3) FA move from the bilayer into the aqueous phase. This process is limited by the rate of desorption, or release of FA into the cytosol (step 3 underlined above), as rates of desorption of most FA were found to be slower than those of adsorption to the membrane (step 1) and flip-flop (step 2) [62]. Proteins associated with transmembrane transport of FA are thus classified as flippases (if they mediate movements from the extracellular phase towards the cytosolic face of the membrane), floppases (if the movement is in the opposite direction) and scramblases (which can transport in either directions but, unlike the other two, are ATP-independent) [59].

While the biochemistry of lipid transporters is subject of much ongoing research and discussion, and details about specific flippases and floppases are beyond the scope of this review, some of the most well-known proteins that operate in this process are type 4 subfamily (P4) of the P-type ATPases. Those translocate aminophospholipids and cardiolipin from the exoplasmic to the cytosolic leaflet; and ATP-binding cassette (ABC) transporters, which transport phospholipids and sphingolipids outwards. ATP-dependent transporters have been reviewed in depth elsewhere [63].

Controversy exists on the importance of flip-flop on the rate of FA passage through the plasma membrane. Flip-flop is largely dependent on the specific physicochemical properties of each FA, which determines the energy barrier for passage through the plasma membrane: a considerable portion of membrane FA are protonated at physiological pH, and would have low energy barrier for transmembrane crossing, compared to fatty acid anions [64]. This has been demonstrated through *in vitro* models, where protonated FA rapidly diffuse across the plasma membrane, by initial desorption from albumin and subsequent flip-flop through the leaflets [65]. Some dependence on the physiological conditions of the organism seems to be at play: pathophysiological conditions have been reported to increase the LCFA:albumin ratio to over 3.0. When said ratios are observed, the concentration of FA unbound to albumin increases, tipping the scale towards transmembrane diffusion; at lower ratios, protein-mediated transport becomes crucial [66]. Translocation across the plasma membrane likely requires a FA translocator in actual biological contexts [61].

#### Fatty acid translocase CD36

The CD36 appears to be the most important FA translocator [67, 68]. A membrane-embedded protein, CD36 is expressed in a variety of cells and tissues [69], and its extracellular domain presents strong binding affinity to a wide range of FA, and even oxidized LDL [70]. Still, the mechanism by which CD36 affects FA uptake is unclear [66], but its activity may also reduce FA “flop”, by maintaining intracellular FA concentrations, thus increasing overall intracellular utilization [71]. In general, however, it is evident that CD36 plays an important role in the overall fate of FA and their concentration within a cell, its activity being tightly regulated by its translocation from endosomes to the plasma membrane (which renders it active) and gene transcription in response to insulin levels, as proposed by Glatz and Luiken [67].

Regulation of CD36 occurs at a transcriptional and translational level, and is mediated chiefly by the transcription factors CCAAT/enhancer-binding protein α (C/EBPα) [68] and PPAR [69]. Fatty acids are strong regulators of the transcription of CD36 in various bovine cell lines [72, 73], likely via PPAR [5, 73]. Based on the above data, it is likely that transcription of CD36 is regulated by the availability of FA in the intestine likely augmenting the absorption of FA when those are increased into the diet [1]. Absorption of macronutrients increases from pregnancy to lactation as observed for amino acids [74]; however, data comparing intestinal FA absorption during the dry or lactating period in dairy cows are not available. Interestingly, in both monogastric and ruminant animals increasing fat in the diet boosts the absorption of FA by the intestine [37, 75, 76]. Thus, it is possible that the increased FA absorption is driven by changes in the expression of main proteins involved in this process, chiefly CD36.

The translocase CD36 appears to be the most important for intestinal FA absorption, at the least in the proximal segment of the small intestine, as demonstrated in intestinal cells isolated from CD36 null mice where FA uptake was reduced by 50% while cholesterol uptake was reduced by 60% compared to wild-type cells [77]. The same mice have defective FA intestinal absorption, TAG synthesis in enterocytes, and intestinal lipoprotein metabolism as observed *in vivo* [78, 79]. Besides FA absorption, CD36 also plays additional functions in the intestine, including a role in controlling the intestinal immune reaction, the microflora, and the FA-induced release of cholecystokinin and secretin by enteroendocrine cells [39, 51, 80].

Although specific studies on CD36 in the intestine of dairy cows are not available, recent RNAseq data became available via the repository NCBI GEO on whole transcriptome of jejunum, liver, and mammary tissue of lactating dairy cows [54]. Those data revealed that the transcription of *CD36* in dairy cows is ~ 10-fold more abundant in jejunum than liver (Fig. 1), supporting an important role of this translocase in the jejunum.

#### Scavenger receptor B1

The SR-B1 (coded by *SCARB1* gene) was originally demonstrated to be expressed in enterocytes in rat where it participates in cholesterol absorption [81]. It appears to play also a role in intestinal absorption of FA or even TAG, as demonstrated in mice over-expressing *Scarb1* and tracing absorption of [^3^H]-triolein [82]. It has been also proposed that SR-B1 might be implicated in the endocytosis of lipids by the enterocytes [82] although this still need be proven. Furthermore, SR-B1 is also involved, together with CD36, in absorption of lipophilic vitamins [83].

The SR-B1 has received little attention among researchers in dairy cows and, to our knowledge, none on its role in lipid absorption by the intestine. The SR-B1 has been associated with marbling in beef animals [84], with dominant follicle formation and luteinization [85, 86], and with the content of carotene in milk [87]. Its expression is induced by somatotropin in liver [88] and by gonadotropin release hormones in the granulosa cells [86]. The increased expression of *SCARB1* by growth hormone is of interest, considering the increased concentration of this hormone early post-partum [89]. In support of its role in bovine intestine, its mRNA is relatively abundant in jejunum and at level similar to the liver in lactating dairy cows (Fig. 1).

#### Membrane-associated fatty acid binding protein

The FABPpm is a membrane-bound protein present in the mitochondria and plasma membrane that is structurally identical and coded by the aspartate aminotransferase 2 gene (*GOT2*) [42]. FABPpm plays important roles in the import of FA in intestine, muscle, and liver [90, 91] by working in concert with CD36 [42, 91, 92]. However, little information was generated on this protein in monogastric and none exists for ruminants. In dairy cows the transcription of *GOT2* in intestine is abundant (Fig. 1). Interestingly, in rat overexpression of FABPpm *in vivo* increased transport and oxidation of FA and formation of phospholipids in sarcolemma but did not increase formation of TAG [91]. A role for this protein in FA absorption appears to be not fully proven [93].

#### Fatty acid transport proteins

The FATP (or soluble carrier protein 27A; *SLC27A*) play two simultaneous roles in FA uptake: membrane translocation and activation to acyl-CoA for down-stream metabolic pathways [94]. Upon conversion to acyl-CoA thioesters, FA cannot escape the cell [95]. There are six isoforms of the SLC27A, with the *SLC27A4* (or FATP4) being in monogastrics the main SLC27A expressed in the intestine [96]. In bovine jejunum, all isoforms appear to be expressed, with *SLC27A2* followed by *SLC27A4* and *SLC27A5* being the most abundant (Fig. 1). The role of this transporter in intestine for the FA absorption appears to be however dispensable [93].

#### Other systems possibly involved in enterocyte absorption of fatty acids

##### Caveolin 1

Caveolins are proteins playing a major role in cellular endocytosis and signalling [97, 98]. Caveolins are known to play a major role in transcytosis, endocytosis, and exocytosis but also for intracellular trafficking between various organelles, including nucleus where they play important roles for transcriptional regulation [98]. The discovery of a role of caveolins in enterocytes uptake of FA is quite recent [53], with a role in apical absorption of FA but also in chylomicron formation (see below). Among the various caveolins, caveolin 1 is known to play a major role in import of FA in enterocytes [99] and its function is independent from CD36 [100]. However, CD36 translocation is regulated by caveolins, chiefly by caveolin-1 [101]. This suggests an inter-regulatory mechanism between proteins in charge of facilitating transmembrane FA crossing and those involved in the shuttling of FA inside the cytoplasm (such as caveolins) [100].

Approx. 15% of the albumin bound ^3^H-oleate was observed to be absorbed by mouse enterocytes via caveolin-mediated endocytosis [99]. A recent study carried out in zebrafish and mouse challenged the prior data on the role of caveolin 1 to import FA from the lumen into the enterocytes [102]. In that study caveolin 1 was found to be localized in the basolateral plasma membrane (i.e., associated with the submucosae that is facing the circulation) rather than apical (i.e., facing the lumen), supporting a role of caveolin 1 in either export of FA from enterocytes to the circulation or having functions in the regulation of insulin signalling in the intestine. Mice with intestinal caveolin 1 knockout had decreased level of cholesterol and increased non-esterified fatty acids (NEFA) in circulation, likely not associated with dietary cholesterol or FA absorption [102].

##### Endocytosis and the role of alkaline phosphatase

Intestinal cells are also able to perform endocytosis of relatively large particles, including exosomes [103], other macromolecules [104], or polymeric micelles [105] via protein-mediated or protein-independent systems [106] indicating this being an additional mechanism for intestinal absorption. Clathrin-dependent type of endocytosis of fat has been reported in mouse *in vitro* model of enterocytes [107]. The clathrin-coated vesicles once inside the cytosol are stored into the enterocytes and processed for exocytosis via lipoprotein formation.

Alkaline phosphatase plays a critical role in FA endocytosis [107]. This enzyme also regulate CD36 activity via phosphorylation in the intestine [75]. Alkaline phosphatase appears to play a regulatory role of FA absorption in intestine, as knockout mice have higher transport of fat droplets through the intestinal epithelium [108]. Interestingly, as for the CD36, also the alkaline phosphatase is associated with lipid draft via glycosylphosphatidylinositol (GPI)-anchored [75]. The GPI are glycolipids that bind the C terminus of proteins tethering them to the plasma membrane. It has been previously reported in mammary tissue a large increase in importance of the GPI biosynthesis during lactation [109], where also increase in transcription of proteins involved in FA import was observed, including CD36 [110]. The gene coding for intestinal alkaline phosphatase (*ALPI*) is expressed only in jejunum in dairy cows (Fig. 1). The above data appears to support a role of alkaline phosphatase in intestinal FA absorption in dairy cows.

### Intracellular transport and activation of absorbed FA

In mammals, the large majority of LCFA are re-esterified into TAG and secreted via chylomicrons that enter the lymphatic system; however, SCFA and MCFA instead enter into the portal vein [111, 112]. The enterocyte processing and transport of those is poorly characterized, especially in ruminants but their absorption and utilization for metabolic purpose is more efficient that LCFA, as observed in monogastrics [112].

#### Activation of fatty acids

Activation of FA into acyl-CoA are necessary for metabolic utilization via TAG synthesis or oxidation [113]. Besides the SLC27A proteins, a major role in activating FA in mammals are the long-chain fatty-acid-coenzyme A ligase (ACSL). The various FATP also interact with ACSL to activate LCFA, as observed in mouse adipocytes [114].

In monogastrics, the acyl-CoA synthetase long chain family member 5 (*ACSL5*) appears to be the major acyl-CoA synthetase in intestine, among the 13 identified isoforms [93], despite being localized in the inner membrane of the mitochondria [115]. The intracellular localization of ACSL5 appears to be a big constraint in the activation of FA for TAG synthesis in the intestine. It is likely that other ACSLs play a role in activating FA for TAG synthesis in the intestine, as also previously proposed to explain the lack of any effect on TAG synthesis in enterocytes after *Acsl5* knockout in mice [93]. In mouse jejunum, *Acsl5* and *Acsl1* were the only expressed isoform with *Acsl5* being > 20-fold higher expressed than *Acsl1* [116]. In bovine, *ACSL1* and *ACSL5* have similar transcription level and are the most abundant ACSL isoforms in jejunum (Fig. 1). The former is also the most abundant ACSL isoform in mammary tissue [117] (Fig. 1) where it likely plays a major role in activating FA for TAG synthesis [110]. It is possible that also in intestine of dairy cows ACSL1 plays the same role.

#### Fatty acid binding proteins

Once the LCFA enter the cytosolic side of the enterocyte membrane, due to their insolubility in the aqueous cytosol they are bound to fatty acid binding proteins, specifically FABP2 (a.k.a., intestinal FABP) and FABP1 (a.k.a., liver FABP) [118, 119], two of the 9 known FABP isoforms [119]. In jejunum of dairy cows most of the FABP are transcribed with the highest mRNA abundance detected for *FABP1* and *FABP2* with the former being > 3-fold more abundant (Fig. 1). Those FABPs transport the FA to esterification, metabolic utilization, or control of gene transcription via PPAR as previously reviewed [118].

The FABP2 transports only FA while FABP1 transports also acyl-CoA (i.e., activated FA) that can enter oxidation or TAG synthesis and can transport FA into the nucleus for activation of PPAR, particularly PPARα [118, 120]. Data from monogastric animals indicate a lower affinity (between 2 to > 20-fold) for UFA vs. SFA for FABP2 while for FABP1 the affinity for UFA is only slightly lower compared to SFA [119, 121]. The FABP1 can bind two FA with a similar affinity between the two sites for SFA but the internal site, the first to bind FA, has > 10-fold higher affinity for UFA vs. SFA [121]. The dissociation constants are in nmol/L ranges [120, 121].

FABP1 transfers FA to and from plasma membranes via an aqueous phase diffusion while other FABPs, including FABP2, by interacting directly with the negatively-charged plasma membranes by the net positively charged Lys residues [120]. FABP2 presents a narrow ligand pocket and strictly binds only LCFA [120] while the FABP1 has a larger ligand pocket and can bind several other lipid molecules besides LCFA, including acyl-CoA [122]. Data on binding affinity between various FABPs in bovine are not available; however, FABP1 in bovine has a very similar affinity among various LCFA as human and mouse FABP1 for the first binding site but higher affinity for stearic acid compared to human (but similar to mouse) for the second binding site [123]. The sequence identity of FABP1 between bovine and the other two species is between 76% and 81%, as determined using NCBI-deposited sequences. The FABP1 and FABP2 carry the LCFA toward the ER for the synthesis of TAG [118]; however, it has been proposed that FABP1 works as reservoir of FA or acyl-CoA in the intestine and can carry FA toward oxidation [124].

#### Acyl-CoA binding protein

The acyl-CoA binding protein (a.k.a., DBI diazepam binding inhibitor, coded by the *DBI* gene) specifically binds and transports medium- and long-chain acyl-CoA [113, 125]. Its function in the intestine is still unclear; however, it might be the main intracellular carrier of acyl-CoA for TAG synthesis [124]. It is generally highly expressed in tissues with high TAG and cholesterol synthesis [125]. Abundance of *DBI* is almost at par with *FABP1* and *FABP2* in intestine of dairy cows (Fig. 1), supporting a role in FA transport in intestine in ruminants.

#### Inhibition of feed intake by dietary FA: possible role of enteric FABP1 and free fatty acid receptors

Supplementation of fat either in the diet or infused in the abomasum often decrease feed intake in dairy cows, especially if UFA are used. The effect was partly explained by a higher induction of intestinal cholecystokinin release, an appetite depressant, by UFA compared to SFA [1]. The increase in circulating NEFA following FA supplementation can also depress appetite through the hepatic oxidation [126]. The above however cannot fully explain the decreased in feed intake.

The role of FABP1 in directing the FA toward intestine catabolism is somewhat peculiar. The oxidation of FA in intestinal cells is minor, as observed *in vitro* in humans [127]; however, it might play a role in regulating feed intake via the vagal nervous system, as previously proposed [128], somewhat similar to the hepatic oxidation theory of the control of feed intake proposed by Allen et al. [126]. The fact that FABP1 is the most abundant FABP in intestine of dairy cows (at the least at transcriptional level), that carries FA towards oxidation, and that can bind two FA (thus, spike in FA absorbed by the intestine are likely taken up by FABP1 compared to FABP2, that can only bind one FA), provide some support for the theorized role of intestinal FA oxidation as an additional way for FA to control appetite. However, this needs to be proven.

Recently, it has been demonstrated that activation of the free fatty acid receptor 1 (FFAR1, a.k.a. G protein-coupled receptor 40) in the enterocytes plays an important role in reducing appetite in rodents [129]. The FFAR1 is activated by LCFA in monogastrics [130] and ruminants [131]. In the latter, the FFAR1 is activated by UFA but not C16:0 [131]. Transcription of *FFAR1* is virtually absent in the jejunum, liver, and mammary tissue of dairy cows while the transcript of other FFAR, including *FFAR4* (a.k.a., GPR120), is detectable in the jejunum, although at relatively low mRNA abundance compared to other transcripts in this tissue (Fig. 1). Transcription of various FFAR genes in several adipose tissue depots, liver, muscle, and mammary tissue in dairy cows was measured [132, 133]. The authors detected an overall low expression of the FFAR genes but with a greater transcription in adipose tissue compared to liver or mammary tissue, suggesting a greater role in the former compared to mammary tissue and liver. In monogastrics, FFAR1 plays a role in CLA-induced fatty liver but it is important to prevent CLA-induced inflammation and insulin resistance in the brain, as observed in Ffar1-knockout mice [134], indicating somewhat tissue-specific effects of FFAR1. The FFAR4 is activated by MCFA and LCFA in monogastrics and can regulate appetite by inducing release of cholecystokinin [130]. To our knowledge, there are not studies in ruminants about the role of FFAR1 and FFAR4 in intestine and control of feed intake.

### Triglycerides synthesis

The enzymatic pathways involved in the synthesis of TAG in the intestine of monogastric species has been previously reviewed in detail [93]. There are two pathways for the synthesis of TAG in the intestine: the monoacylglycerol and the glycerol-3-phosphate (G3P) pathways. Both pathways are operative in monogastric animals while the second is predominant in ruminants likely due to the negligible amount of absorbed monoacylglycerol [8], as observed in sheep [135].

As previously described for the synthesis of TAG in the mammary tissue [110], the first step for the G3P pathway is the formation of the lysophosphatidic acid by the binding of acyl-CoA [118] with glycerol-3-P by the glycerol-3-phosphate acyltransferase enzyme (GPAT). The second acyl-CoA is then added to the *sn*-2 position by the 1-acylglycerol-3-phosphate-O-acyltransferase (AGPAT) to form the phosphatidic acid. The P group is removed by lipin (LPIN) to form diacylglycerol (DAG). The last acyl-CoA is added to the DAG by the diglyceride acyltransferase (DGAT, specifically DGAT1 for secreted lipoproteins and DGAT2 for stored lipid TAG-rich lipid droplets [93]).

Due to the large lipophilic property of the TAG, this is formed in between the two leaflets of the ER membrane. As the TAG are inserted into the ER membrane, a lipid droplet is formed. If there is active translation of the apolipoprotein B48 (apoB48) by the ribosomes docked to the ER, the growing lipid droplets lipidate the apoB48 forming the prechylomicron particle. This is exported to the Golgi [136], otherwise, it bud off entering the cytosol where it is stored in the apical part of the enterocyte forming a temporary “storage” but it is then used for the formation of chylomicron [93, 136, 137]. This is important in monogastric, especially human, where there is intermittent feeding of relatively high-fat diet with moments of large intestinal absorption followed by moments of low absorption; in ruminants, where the flow of digesta is quite constant and the level of fat in the diet is low, we expect the formation of storage lipid droplets in enterocytes being rare.

All the enzymes responsible for the TAG synthesis present several isoforms. In intestine, *Gpat3*, *Gpat4*, *Agpat1*, *Agpat2*, *Agpat3*, and *Lpin3* are the most abundant or important among their respective isoforms in monogastrics [93]. Transcript abundance of the various isoforms of the TAG-synthesis related genes in dairy cows has been determined for the mammary tissue [117]. Transcript abundance of the various isoforms of the TAG-related genes in mammary, liver, and jejunum are remarkable similar between the three tissues, with few exceptions (Fig. 1). All three tissues are known to be involved in TAG synthesis in monogastrics; however, in bovine the liver is not considered an important site of TAG synthesis [138] except early post-partum in dairy cows, when NEFA significantly increase in circulation [139]. Thus, the high abundance of those isoforms in liver is peculiar and the reason for that is unclear.

Among GPAT, the transcription of *GAPT3* and *GPAT4* are the most abundant in the jejunum of dairy cows (Fig. 1). Those GPAT isoforms are localized in the ER [140]; however, also the mitochondrial isoform (*GPAM,* a.k.a. *GPAT1*) has a similar abundance (Fig. 1). Although the subcellular location of this isoform is in the mitochondria, the active enzymatic domain faces the cytosol [140]. The mitochondrial location of GPAM appears also to play a pivotal role in TAG synthesis by competing with utilization of FA for oxidation by carnitine-palmitoyl transferase 1 (CPT1) [140]. Among GPAT, GPAM has large preference for saturated LCFA and its transcription is regulated by SREBP1 while *GPAT3* is regulated by PPARγ and *GPAT4*, initially classified as *AGPAT6*, appears to have not preferences among LCFA but its role in TAG synthesis remains undetermined [140]. Interestingly, transcription of PPARα and PPARγ and of the two SREBF isoforms is abundant in jejunum of dairy cows (Fig. 1). An important role for *GPAT4* (reported as *AGPAT6*) and GPAM in TAG synthesis in dairy cows is also supported by this GPAT isoforms being the most abundant in lactating mammary tissue, where synthesis of TAG is predominant [110].

Among AGPAT, the most abundant isoforms in jejunum of dairy cows are *AGPAT1* and *AGPAT5* followed by *AGPAT3* (Fig. 1). All those isoforms are located in the ER and *AGPAT3* is known to be regulated by PPARα in monogastric animals [140]. As for jejunum, also in mammary gland the *AGPAT1* and *AGPAT3* are the most abundant AGPAT isoforms (Fig. 1) [117].

A recent publication reviewed the most up-to-date information about LPIN isoforms in monogastrics [141]. According to data reported in that publication, the relative abundance between isoforms in intestine is to some extent similar to the one detected in jejunum of dairy cows (Fig. 1). Among LPIN isoforms, *LPIN1* has been the most studied. The lipin proteins resides into the cytosol, but they are translocated into the ER when FA increase in the cytosol; interestingly, in monogastrics the UFA, but not SFA, induce translocation of lipin into the ER. Nuclear localization and a role in FA activation by PPAR were determined for lipin1 and lipin2. In bovine mammary tissue, among LPIN isoforms, only transcription of *LPIN1* was increased through lactation, supporting a role of this lipin in TAG synthesis in this tissue [117].

## Formation and release of intestinal lipoproteins

The study of lipoproteins in bovine was intense several years ago as previously reviewed [37, 111, 142]. It has been debated in the past about the production of very-low density lipoproteins (VLDL) instead of chylomicron in intestine of ruminants, due to the high proportion of VLDL in the lymph [37, 111]. The high proportion of VLDL is due to the relatively low amount of dietary FA in ruminants; however, higher amount of fat in the diet increases production of chylomicrons, as well higher proportion of UFA are inserted into chylomicrons, as previously reviewed [111].

In bovine, lipoproteins have been well-characterized and, contrary to monogastrics, the HDL compose the large majority (> 80%) of circulating lipoproteins [37]. Little advance in our understanding of lipoproteins formation and metabolism have been made in dairy cows in the last decade and, to our knowledge, none on the synthesis of intestinal lipoproteins. The emergency and intensification of lipid-related diseases in humans has prompted an intense study of the molecular aspects of the lipoprotein metabolism, including the intestinal lipoproteins. Thus, most advances on molecular aspects of the lipoprotein metabolism have been made on human and other monogastric animals. Several excellent reviews have been published in this regards [42, 136, 137, 143]. In this section, we provide a summary of the most up-to-date model on intestinal lipoprotein metabolism based on the above reviews and provide data obtained from dairy cows when available.

### ApoB and the synthesis of chylomicrons

There are several apolipoproteins secreted by the intestine, but the apoB48 is the one essential for the formation of chylomicrons [136]. The apoB48 is coded by the *APOB* gene in all mammals, including bovine. This gene in liver codes for the apoB100 protein; however, in the intestine the *APOB* mRNA editing complex (APOBEC) inserts a stop codon at approx. 48% of the full-length sequence of the *APOB*, removing the C-terminus that binds light-density lipoprotein receptor (LDLR). The *APOBEC1* and *APOBEC3H* are the two APOBEC most transcribed into the jejunum (Fig. 1). Of the two, only APOBEC1 is known to be involved in apoB editing and be expressed in intestine and liver in mice [144]. Contrary to mice, bovine *APOBEC1* is not expressed in the liver (Fig. 1). There is not information on role of APOBEC3H on lipoprotein synthesis. The *APOB* is the most transcriptionally abundant apolipoprotein in jejunum of dairy cows; however, its mRNA abundance is > 5-fold lower compared to the liver (Fig. 1).

The *APOB* mRNA is initially translated in the cytosol; however, as the first 27 codons are translated the process pauses. The first 27 amino acids of the sequence are used as a signal to translocate the mRNA-ribosomal complex to the translocon of the ER, where the rest of the protein is translated in the ER lumen and TAG and phospholipids are transferred to lipidate the nascent protein by the microsomal triglyceride transfer protein (MTTP). The MTTP is essential for the formation of chylomicrons or VLDL in the intestine and liver. Interestingly, *MTTP* transcription is almost 2-fold larger in the jejunum compared to liver in dairy cows (Fig. 1). The nascent apoB48 is immediately degraded if not properly lapidated. The presence of TAG is essential for the formation of chylomicrons or VLDL (compositing 67–88% of lipids in lipoproteins in bovine), as well the presence of phospholipids (8–20%) and cholesterol (2–10%) [37].

### Importance of phospholipids and cholesterol for the synthesis of chylomicrons

#### Phospholipids

The importance of phospholipids for synthesis of lipoproteins in dairy cows is supported by the fact that dietary supplements that help increasing synthesis of phospholipids, such as choline [145–147], improve the formation of VLDL in bovine hepatocytes [148] and/or decrease accumulation of fat in the liver [145, 147]. Phospholipids in intestine might not be limiting for ruminants, especially if grazing, considering the large amount of them present in chloroplasts. However, a large amount of phospholipids derives from the intestinal hydrolysis of biliary phospholipids, especially phosphocholine that represents the majority of bile lipids in ruminants [8].

#### Cholesterol

Cholesterol is also essential for the formation of chylomicrons. It has been reported previously that addition of cholesterol in the diet of preruminant calves increases the production of chylomicrons [111]. Cholesterol is however very low in the diet of dairy cows indicating that the cholesterol has to be almost completely synthesized by the animal [8]. In ruminants, the intestine appears to be the major site of cholesterol synthesis [8] as demonstrated in goats [149]. Adipose tissue and intestine accounted for the large majority of *de novo* synthesis of cholesterol using acetate and glucose, with liver accounting for < 5% in goats [149]. Despite the large importance of intestine and adipose tissue on cholesterol synthesis in ruminants, recent studies on cholesterol metabolism in dairy cows have concentrated on the liver [150, 151] with no studies on intestine or adipose tissue.

In monogastric animals, the cholesterol used for chylomicron or VLDL synthesis in enterocytes mainly derive from the biliary cholesterol reabsorption via the cannalicular sterol transporter Niemann-Pick C1-like 1 (NPC1L1). This can be an important way to provide cholesterol for enterocytes of dairy cows, as supported by the high mRNA abundance of the *NPC1L1* in the jejunum, especially compared to liver and mammary tissue (Fig. 1). Recent data in monogastrics indicate the existence of a reverse transport of cholesterol from blood to intestine via the LDLR with consequent endocytosis of hepatic-derived apoB-containing lipoproteins (apparently different than VLDL) that are made to eliminate excess free cholesterol from the liver [83]. This can be an additional pathway for the availability of cholesterol in enterocytes. In support of this, the transcription of LDLR is > 4-fold larger in jejunum compared to liver in dairy cows (Fig. 1).

Although all the above means to provide cholesterol for chylomicron synthesis are possible also in ruminant, the major contributor of cholesterol for lipoprotein synthesis in enterocytes is the *de novo* synthesis of cholesterol by ruminant enterocytes. As observed earlier [8], cholesterol in plasma typically increases as fat is supplemented in the diet of dairy cows [152–154] and decreases around parturition with a constant increase during the first two months postpartum [151, 155]. This change has a similar pattern as the typical increase in feed intake of the dairy cows post-partum [156].

It is unclear if an active regulation of cholesterol synthesis in the intestine exists. The main regulators of genes involved in cholesterol synthesis in monogastrics are the SREBP1 and SREBP2, with a more prominent role of SREBP2 [157], including for the intestine [158]. Change in mRNA abundance of *SREBF1* and several down-stream targets related to cholesterol synthesis coordinately increase in liver of post- vs. pre- partum dairy cows, indicating an increased hepatic cholesterol synthesis [150]. These data appears to support the apparent increase in hepatic synthesis of apolipoproteins post-partum that can be compromised by inflammatory-like condition, since concentration of total cholesterol in blood can be used as index of liver activity in early post-partum cows [155, 159]. The lower amount of cholesterol early post-partum might also indicate a lower capacity of the intestine to produce chylomicrons and, thus, likely a lower ability to use supplemental fat compared to cows in more advanced stage of lactation. Unfortunately, data on the FA absorption capacity of the intestine of dairy cows during the various stages of lactation are not available. However, two pieces of evidence support a lower intestinal FA absorption in early vs. late lactating dairy cows. The first piece of evidence is that > 65% of the circulating TAG are of gut origin in ruminants [111] and there is a lower level of TAG and phospholipids in plasma (mostly present in chylomicron remnant, VLDL, and light density lipoproteins) in cows during early vs. more advanced stages of lactation [151, 155]. The second piece of evidence is the lack of response of cholesterol concentration in blood in cows supplemented with large amount of palmitic acid (4% dry matter intake ) during the first week post-partum while a strong response is observed in cows at 3 and 7 weeks into lactation [152].

### Additional apolipoproteins present in chylomicrons

Once the prechylomicrons containing apoB48 are formed into the ER, they contain also apoA-IV and they bud-off from the ER and are transported to the Golgi by a combination of a chylomicron transport vesicle that implies several proteins, including CD36 and several FABP, and the COPII coatomer transport system [136]. The prechylomicrons then fuse with the *cis*-Golgi thanks to the COPII system [160]. In the Golgi, prechylomicrons acquire more apoA-IV and, by the activity of the MTTP, more TAG. The final mature chylomicrons containing apoB48, ApoA-IV, cholesterol ester, TAG, and phospholipids are then secreted via exocytosis facilitated by a N-ethylmaleimide-sensitive factor attachment protein receptor (SNARE) from the basolateral membrane of enterocytes into the lymphatic system [136].

Among apolipoproteins, apoA-IV is essential for the formation and secretion of chylomicrons but has also several other functions, including regulation of lipid metabolism, satiety, and size of secreted chylomicrons (larger when apoA-IV is knockout), at the least as observed in monogastric animals [161]. One of its important function, especially for the apoA-IV produced by the liver, is the formation of HDL. This is also achieved by activating the lecithin–cholesterol acyltransferase, an enzyme present on HDL with an essential role in the conversion of free cholesterol into cholesteryl ester that is then accumulated into the core of HDL, essential for the formation of mature HDL [136]. Although the HDL are the majority of lipoproteins in circulation in dairy cows [37], the significance of this is still unclear. The importance of apoA-IV for the formation of chylomicrons in the intestine of dairy cows is supported by the mRNA abundance, which is similar to the one observed in liver (Fig. 1).

The chylomicrons acquire additional apolipoproteins while circulating in the lymphatic system and blood. However, several of those are expressed also in the intestine, such as apoA-I, apoC-II, apoC-III, and apoE (Fig. 1). Bauchart provided a list of apolipoproteins present in bovine lipoproteins [37]. The list included most of the apolipoproteins detected in monogastrics as reported recently by Ramasamy [136], except for apoE, apoA-II, and apoA-V which were only reported to be present in chylomicrons of humans by Ramasamy. Apolipoproteins are mostly synthesized between liver and intestine, with the exception of apoC-I, which in monogastrics is synthesized only in the liver [136]. In dairy cows, RNAseq data indicate that all apolipoproteins, except apoA-V and apoC-IV, are transcribed in the jejunum of dairy cows but, beside transcription of all apolipoproteins in liver, also mammary tissue can transcribe genes coding for apolipoproteins, such as apoE (Fig. 1). Among the apolipoproteins, it is important to highlight the function of other apolipoproteins present in chylomicrons. The apoA-V and apoC-II are activators of lipoprotein lipase (LPL) [162]. ApoC-III is an inhibitor of LPL and VLDL and LDL clearance by the liver [162]. ApoE activates LDLR [163], important for the removal of chylomicron remnants by the liver [136].

### Lymphatic transport of chylomicrons

In general, intestinal chylomicrons and VLDL released by the basolateral membrane of enterocytes enter the lymphatic system via the lacteal system, as previously reviewed [164]. However, it has been determined in preruminant calves that between 20% to 67% of chylomicrons and between 50% and 80% of VLDL produced by the intestine are released into the portal vein, reaching a higher proportion at peak absorption after providing the milk to calves once a day [165]. This is likely due to the coagulation of milk in the abomasum of pre-ruminant calves and the consequent slow passage rate of fat in the intestine [37]. The importance of portal vein in the transport of intestinal chylomicrons and VLDL in adult ruminant is not known but could be important when the passage rate of lipids in the intestine is slow, as previously argued by Bauchart [37].

It is still unclear the mechanism for the entrance of chylomicrons into the lymphatic system via lymphatic endothelial cells. So far, two mechanisms have been proposed and supported by data: the flow through opening of intercellular gaps and transcytosis. Also the mechanism for the flow of the chylomicrons inside the lymphatic system is not fully characterized, but it appears to be mainly due to the peristaltic activity of the intestine [164]. Interestingly, the flow into the intestinal lymph was significantly stimulated by injecting oil in abomasum and duodenum of lambs [166] but was not affected by feeding cows with oil [76]. In the latter study, feed deprivation of the animals significantly affected lymphatic flow.

The flow of the intestinal lymphatic system in cows has been measured to be 2–3 L/h (or 30–50 mL/min). Labelled FA appear in the lymph ca. 1 h after injecting oil in the abomasum with a significant increase in TAG absorption ca. 3 h after injection of oil in abomasum or duodenum in lambs. In the same study, when the oil was injected into the rumen of sheep or provided via stomach tube in lambs, the radioactivity in the intestinal lymph was observed after 6 h post-injection [166]. Similarly, in dairy cows significant absorption of TAG in the lymph was observed between 7 and 16 h post-feeding, with maximum absorption detected around 12 h post-feeding [76]. In the same study, it was calculated the rate of absorption of TAG into the lymphatic system: TAG were absorbed to a rate of 20–40 g/h while phospholipids and cholesterol at rate of 4–6 and 1–2 g/h, respectively.

The above study [76] was performed in 1966 using Jersey cows. Nowadays, cows have higher feed intake; thus, the rate of absorption is likely larger. However, the Jersey cows appears to have a higher passage rate compared to Holstein cows [167]. Assuming the above absorption rate, a Holstein cow eating 20 kg of dry matter (DM)/d of a diet with 5% fat (i.e., 1 kg of fat) it would absorb the dietary fat in about 30 h. From the nutrigenomic point of view those data are of importance when considering that the concentration of fed FA into the plasma has to achieve the desired effect (i.e., change in the transcriptome) [4].

With the above data, it is possible to estimate the amount of dietary FA needed to achieve an effective dose to optimize their nutrigenomic effect. For instance, when considering palmitic acid with a hypothetical maximal nutrigenomic effect achieved at or above 100 μmol/L, feeding 500 g/d of pure palmitic acid should provide around 0.01 g/min (or 50 μmol/L/min) of C16:0 in the plasma. The calculations were based on 40 g/h of dietary TAG transported into the lymphatic system and a basal concentration in plasma of 20 μmol/L of C16:0 [4] in a dairy cow (600 kg BW) receiving around 0.5 kg of fat from forages containing 30% palmitic acid. However, recent data indicated that concentration of dietary FA in blood is probably not a correct approach, because it does not account for the local increase in concentration by the activity of LPL on lipoproteins [168].

## Utilization of dietary fatty acids by peripheral tissues

### Catabolism of chylomicrons and other lipoproteins

The clearance of TAG in chylomicrons is very fast, especially in ruminants. The turnover rate of FA in plasma of lactating cows is between 2 and 9 min [8, 111]. In an elegant study performed approx. 40 years ago, Bergman and collaborators injected chylomicrons obtained from the intestinal lymphatic system of donor sheep where palmitic acid labelled with ^14^C and ^3^H was infused into the lymphatic system of the duodenum in recipient sheep [169]. It was determined that the turnover rate of TAG in chylomicrons after termination of lymph infusion was very rapid, between 8 and 9 s. In the same study, it was detected that around 50% of TAG hydrolyzed from the chylomicrons were found circulating as NEFA, and that the peripheral tissue utilizes 11–15% of TAG in chylomicrons per each circulation while liver utilizes only about 2% per circulation, with a final utilization of 10% by the liver of infused TAG present in chylomicrons. In human chylomicrons disappear from circulation within 12–14 h after a meal [136].

Lipids included in lipoproteins can enter the cell in one of two main ways: through endocytosis of the entire lipoprotein molecule and eventual intracellular metabolization (mainly liver, but also macrophages, vascular smooth vascular cells, kidney, gonads, and adrenals) [170, 171] or by hydrolysis of the TAG contained within the lipoproteins (mostly for adipose tissue, skeletal muscle, and heart in non-lactating animals and mammary tissue in lactating animals), which occurs at the luminal surface of the capillary endothelial [172].

#### Lipoprotein endocytosis

Chylomicrons are too large to cross the tightly associated endothelium or be taken up via endocytosis and are mainly processed through hydrolysis [172]. The same cannot be said of HDL, where their smaller size allow them to be taken up via endocytosis by various tissues, including liver, macrophages, and kidney [170, 173, 174]; while LDL bind to the LDLR, and are taken up by hepatocytes through clathrin-coated pits [175]. Once the LDL-LDLR are in the endosome, the LDLR detaches from the LDL due to a decrease in pH [176] and the LDL is directed to the lysosomes, where it is subsequently degraded. Chylomicron remnants and VLDL remnants are taken up (mostly by hepatocytes) through a similar process, mediated by several receptors of the LDLR family, namely LDLR, VLDLR, LDLR-related protein 1 [170]. In monogastrics it has been determined that chylomicron remnants clearance by the liver requires the binding of four LDLR via the apo-E [136]. The TAG in VLDL remnant are also further hydrolyzed through hepatic lipase with production of LDL [170, 177]. 

#### Hydrolysis of TAG in lipoproteins by lipases

Hydrolysis of lipoproteins is mediated by the action of lipases [178]. Several lipases act on dietary lipids, chiefly among them are pancreatic lipase that acts on the intestine [179], while endothelial lipase [180], hepatic lipase [181], and most notably LPL [179] act on circulating lipoproteins. The aforementioned act upon TAG present in chylomicrons and VLDL, converting each TAG into a *sn-*2-monoacylglycerol and two NEFA [182].

##### Lipoprotein lipase

The fast rate of utilization of circulating chylomicrons is due to the activity of LPL. The transcription and activity of LPL is the highest in adipose tissue followed by skeletal muscle, heart and lungs, and to a lesser extent spleen, rumen, and small intestine [183, 184]. The lactating mammary gland also presents a larger transcript abundance and activity of LPL [185] (Fig. 1), with a marked increase in its transcription and activity at the onset of lactation [110, 186], possibly induced by prolactin [187]. Interestingly, in adipose tissue prolactin appears to inhibit LPL activity during lactation, at the least in humans [187], to augment availability of FA to the mammary tissue [188]. Transcription of *LPL* in bovine mammary is also affected by diet as observed in dairy goats and cows [189–191].

The LPL activity in bovine is around 1000 mU (i.e., 1 U = 1 nmol/L of released FA/min)/g of tissue in the adipose tissue, around 500 mU/g of tissue in muscle, around 300 μmol/L of FA/h/g of tissue in mammary gland [185], and 1000 nmol/L FA/h/mL of plasma collected from the mammary vein [192].

In monogastrics, the abundance of LPL is tightly controlled by hormones, such as insulin, glucocorticoids, and adrenaline [136, 193, 194]. It has been also considered a PPARγ target gene, but its transcription is not increased by PPARγ agonist in bovine [72] or goat [195]. In bovine mammary tissue its transcription is decreased by milk fat depressing diet and several rumen biohydrogenated UFA, especially *trans-*10,*cis-*12 CLA [196–198].

The LPL is synthesized in various tissues, it is then folded and assembled in a homodimer before being excreted and transported to the endothelial lumen by the glycosylphosphatidylinositol-anchored high-density lipoprotein binding protein 1 (GPIHBP1) [199]. The GPIHBP1 is also essential for the LPL catalytic activity [200]. There are newly-discovered proteins important for LPL activity, as recently summarized [136]. In monogastric animals angiopoietin-like 4 (ANGPTL4) acts as LPL inhibitor [201] and lipase maturation factor 1 is essential for the folding and maturation of LPL [202]. No information exists in dairy cows on the role of those on LPL activity. ANGPL4 is highly expressed in liver and adipose tissue and its transcription is likely regulated by PPAR [5, 203]. A positive association between GPIHBP1 and milk fat synthesis has been detected in bovine [204], likely by promoting the activity of LPL.

The release of FA by the hydrolysis of TAG by LPL has a large increase in concentration of FA locally, likely reaching the desired biological activity of the fed FA, as recently demonstrated *in vitro* in our laboratory [168]. However, NEFA released from the chylomicrons and VLDL by LPL do not completely permeate into the extracellular space of tissues, rather they are taken up by albumin to be part of the circulating NEFA pool. It has been estimated that in ruminants not in negative energy balance, around 60% of circulating NEFA are derived from hydrolysis of TAG present in lipoproteins [111].

A pivotal role in regulating lipoprotein metabolism is mediated by the interaction of LPL with receptors of the LDLR family [205]. Several investigators have reported significant binding affinity of LPL with LDLR, LDLR-related protein 1, VLDLR, and ApoE receptor 2 [206]. The interaction of lipases and the LDLR family seems to involve both lipoprotein endocytosis and hydrolysis: indeed, the LDLR was found to be required for LPL-mediated lipoprotein uptake in mouse endothelial cells [207]. LPL is also transcribed in bovine liver and jejunum, although being about 100-fold lower than in mammary tissue (Fig. 1).

The VLDLR is crucial in tissues where FA are used as fuel, such as skeletal muscle and the heart, playing a major role in the regulation of LPL-mediated lipolysis [208]. This is mostly achieved by VLDLR binding VLDL and bringing the lipoprotein in proximity to LPL for hydrolysis. The VLDLR also aid in the transport of LPL to the endothelial surface and play a role in LPL-mediated endocytosis of lipoproteins, as observed in muscles [208]. In dairy cows, data indicated a coordinated work of VLDLR and LPL in the large uptake of FA from circulating chylomicrons and VLDL by the lactating mammary gland [110, 209].

##### Hepatic lipase and hormone-sensitive lipase

In monogastrics, hepatic lipase (coded by the *LIPC* gene) is an important enzyme for the utilization by the liver of TAG in circulating lipoproteins [181]. Early studies found a markedly low activity of hepatic lipase in the bovine liver compared to monogastrics (~ 15 fold less than in the liver of rats, per gram of tissue) [210]. We are not aware of any additional study on bovine hepatic lipase. Despite that study indicating a lower activity of hepatic lipase compared to monogastrics, the transcription of *LIPC* is relatively abundant in liver of dairy cows and at the least 10-fold higher than *LPL* (Fig. 1). Thus, the functional importance of hepatic lipase in dairy cows, in particular the role in the uptake of dietary FA by the liver, should be further investigated.

The role of hormone-sensitive lipase (HSL, encoded by the *LIPE* gene), originally thought to be solely involved in lipolysis of the adipose tissue in response to increased circulating epinephrine, was found to be expressed in rat mammary tissue, where it plays a role in mammary cell cholesterol esterase activity [211]. *LIPE* is expressed, although at low level, also in bovine mammary tissue (Fig. 1). Interestingly, *LIPE* is upregulated in response to FA (chiefly palmitate and stearate) in an *in vitro* bovine mammary epithelial cell model, which suggests that dietary regulation is a viable avenue [212]. However, the role and importance of mammary HSL in dairy cows needs to be further investigated.

### Transport of FA from endothelial lumen to cells

Upon hydrolysis, some of the released FA are used by the surrounding tissues through interaction with cell surface receptors involved in uptake of lipids, such as CD36 [213], and can be utilized by the cell for fuel or added to the TAG storage in lipid droplets [214]. A portion of the released NEFA is not utilized by tissues, and instead enters blood circulation [214]; since the solubility of most FA in aqueous solutions is below 30 μmol/L [215], circulating NEFA bind to albumin, which acts as a FA transporter in the blood [216]. Albumin-FA complexes are then delivered to the tissues; the complexes can cross the endothelium either through fenestrated endothelium, the discontinuous sinusoidal endothelia of the liver, endothelial clefts, or via endothelium transcytosis through binding with the receptor albondin [217, 218]. Finally, albumin-FA complexes likely bind specific receptors (such as gp60) at the surface of cells allowing the uptake of FA by the cells [216, 219, 220], even by endocytosis of albumin, although the quantitative importance of this is unclear [221].

Free FA (i.e., unbound from albumin) are then imported by the cell through the fatty acid import mechanisms that are described for the enterocyte absorption of FA (described above). The circulation of dietary FA shuttled through albumin allows for delivery of NEFA to tissues where rates of LPL-mediated hydrolysis of lipoprotein is low, and as such, the activity of LPL is a major factor in facilitating and regulating the cellular uptake of FA and other lipids [222], including cholesteryl esters [213].

### Chylomicron clearance and uptake of dietary FA by the liver

The chylomicrons after the TAG have been hydrolyzed by the peripheral tissue become chylomicron remnant [136]. The clearance of those particles by the liver is not fully clear. Based on the review by Ramasamy [136], the TAG remaining in those lipoproteins are further hydrolyzed by LPL and hepatic lipase present in the space of Disse of the liver. Liver of dairy cows expressed both enzymes, as determined by mRNA abundance, supporting a similar role in this species (Fig. 1), although the hydrolysis of TAG present in chylomicron remnants by lipases in the liver of dairy cows is likely lower than in monogastrics [210].

As for other mammals, in dairy cows dietary FA reach the liver mostly through endocytosis of chylomicron remnants. In sheep, uptake of FA derived from TAG present in lipoproteins by the liver accounts for about 10% of the total TAG removed from the blood [169]; thus, a relatively low amount of dietary FA is taken by the liver.

Dietary LCFA can also reach the liver via the hydrolysis of chylomicrons by LPL in the peripheral tissues. This activity releases a large amount of NEFA that are partly taken up by albumin and participate to the circulating NEFA pool. Liver is known to take up around 25% of circulating NEFA and can be catabolized or re-esterified and stored as TAG [223]. In lactating goats, infused palmitic acid is mostly stored as TAG in the liver [224], indicating that the majority of NEFA released by LPL that reach the liver are likely stored as TAG.

## Cellular fate of fatty acids

### Cellular import, intracellular shuttling, and utilization of FA by the liver

Different than monogastric species, the adipose tissue of ruminants accounts for > 90% of *de novo* FA synthesis in non-lactating ruminants, while the liver plays a minor role [138]; thus, most of the FA present in the hepatocytes of dairy cows are derived from the diet and from the lipolysis of the adipose tissue. The FA are imported and activated for metabolic utilization as described in the section titled “Molecules involved in intestinal FA absorption”. The topic of FA metabolism has been reviewed extensively by other authors [1, 3, 225], so here we will only include a brief summary of the main intracellular processes related to FA metabolism. Acyl-CoA thioesters are recognized by multiple proteins in charge of directing FA to the appropriate organelles, such as FABP and DBI [226]. A model summarizing the various steps in FA uptake by the liver and their utilization is available in Fig. 2.

#### Catabolism of fatty acids

The main pathway for FA catabolism is β-oxidation, which occurs, albeit with key differences, in the mitochondria and the peroxisomes [54]. In order to enter the mitochondria, acyl-CoA must be conjugated to carnitine, which binds to the LCFA forming acylcarnitine [227]. This action is catalyzed by CPT1, of which three isoforms are known (CPT1A, CPT1B and CPT1C) [228] with CPT1A being the most abundantly transcribed in intestine, liver, and mammary (Fig. 1). Acylcarnitine then cross the mitochondrial membrane through the action of carnitine acylcarnitine translocase (CACT, coded by the *SLC25A20* gene, Fig. 1). Upon removal of carnitine from acylcarnitine, CACT also returns carnitine to the cytoplasm, where another cycle can begin [227], while the acyl-CoA is oxidized to produce acetyl-CoA for TCA cycle and ultimately production of energy via oxidative phosphorylation, or, if not completely oxidized, ketone bodies production in the liver [226]. While the oxidation of MCFA and LCFA is handled by the mitochondria, very long-chain FA (≥22 carbons) are not substrate of CPT1 and cannot enter the mitochondria. As such they are shuttled to peroxisomes by ATP-binding cassette transporters (ABCD1/2/3) where they undergo incomplete oxidation, are conjugated with carnitine through carnitine O-octanoyltransferase, and are transported to the mitochondria to complete their oxidation [226].

#### Esterification of fatty acids in triglycerides

Besides β-oxidation, intracellular FA can also be esterified into TAG, mainly through the G3P pathway, as described for the enterocytes above. In the final step of TAG synthesis in liver, DGAT1 channels newly formed TAG into VLDL while DGAT2 synthesizes TAG for storage in lipid droplets. Transcription abundance of both DGAT isoforms are very similar in liver (Fig. 1). Finally, lipid droplets can be hydrolyzed, and the resulting FA can be routed into VLDL for secretion, again by DGAT1 [229].

Synthesis and secretion of VLDL from hepatocytes is regulated by rates of synthesis of apolipoprotein B100. Lipid components are added to apolipoprotein B by MTTP [230]; after glycosylation of the apolipoproteins and secretion from the Golgi membrane, VLDL fuse with membrane and are released into the blood.

In liver of monogastrics, exogenous FA are preferentially stored as TAG in lipid droplets, while *de novo* synthesized FA are likely channeled to TAG synthesis for VLDL formation [231], this is also true in ruminants, as most of the 1-^14^C palmitic acid infused into jugular is stored as TAG in the liver of lactating goats [224]. As a consequence of the different fates of *de novo*-synthesized and exogenous FA in liver, animals with lower rates of FA synthesis in the liver display lower rates of VLDL secretion: in ruminants (cows [232] and goats [233]), that is indeed the case. Lower secretion of VLDL in ruminants vs. monogastrics has been known for some time, as reviewed by others more than two decades ago [111, 234, 235]. The liver in ruminants has a low capacity for *de novo* FA synthesis compared to monogastrics where, in the latter, the liver is the main lipogenic organ [138]. The low *de novo* synthesis of FA in the liver can help to explain the low VLDL secretion in ruminants; however, the fundamental reason for the observed difference in VLDL secretion between ruminants and monogastric animals has not been fully elucidated.

Availability of FA in the hepatocyte, while certainly a factor in determining VLDL secretion, is less important than the source of said FA, as is noted in monogastrics, where an increase in plasma NEFA stimulates VLDL secretion in the liver [236]. This is of particular interest in the context of dairy cattle nutrition in the transition from pregnancy to lactation, where a significant increase in circulating NEFA is expected, due to the lipolysis of the adipose tissue in response to the negative energy balance experienced by the animals [223]. However, despite the ability of the liver to decrease hepatic TAG content by increasing VLDL secretion when circulating NEFA are high, an imbalance between FA import and secretion is possible in the peripartum period, which leads to hepatic TAG accumulation [111, 237]. When the accumulation of TAG in liver becomes severe (> 10% DM) the organ is unable to work properly [238]. Moderate and severe fatty liver in dairy cows are associated with several metabolic diseases, negatively affecting reproductive performance and the immune system [239, 240]. Interestingly, cows that are overfed in the dry period display greater accumulation of TAG in the liver post-partum likely due to higher NEFA in plasma, as well as increased transcription of stress and inflammation-related genes compared to cows fed a balanced diet [241]. This is in line with the finding that NEFA are preferentially stored as TAG. On the other hand, if NEFA are mostly re-esterified in the liver, they are less available to affect the transcriptome. However, it has been previously argued that NEFA may be important to control the transcriptome of the liver of dairy cows early postpartum [5]. Results from our laboratory show that NEFA can modulate PPAR, with a marked dose-dependent response [168].

## Regulation of the transcriptome by fatty acids

Fatty acids are bioactive in several ways, including affecting the transcriptome. This is mediated by certain TF that serve as transcriptional regulators. The activity of the TF is modulated either directly via the binding of FA to a ligand pocket and subsequent activation or repression or indirectly. Review about transcription factors that respond to FA in ruminants [4] or monogastrics [225] are available elsewhere. Here we provide an overview and some updated information regarding the role of those TF and their modulation by FA in ruminants in general but more specifically in dairy cows.

### Peroxisome proliferator-activated receptors

A comprehensive review on PPAR in ruminants was published few years ago [5]. The PPAR are transcription factors belonging to the nuclear receptor superfamily. Three isotypes have been characterized: PPARα, highly transcribed in tissues that rely significantly on FA catabolism such as liver, kidney, adipose tissue, and muscle; PPARβ/δ with a widespread transcription pattern; and PPARγ, of which two isoforms are known: PPARγ1, with a widespread transcription pattern, and PPARγ2, detected at high level in the adipose tissue [5].

Ligands of PPAR are primarily LCFA and eicosanoids. These interact with PPAR by directly binding its ligand-binding domain, causing it to form a heterodimer with another nuclear receptor, the retinoid X receptor (RXR). The PPAR-RXR complex subsequently bind the DNA at specific recognition sites termed PPAR response elements initiating the transcriptional cascade [242].

The three PPAR isotypes are primarily involved in transcriptional regulation of genes coding for proteins related to lipid metabolism [5]. PPARα plays a pivotal role in all aspect of the catabolism of FA, including uptake of FA, carnitine synthesis and carnitine uptake into cells, FA transfer into mitochondrion by CACT, and β-oxidation in mitochondria and peroxisomes, especially in the liver [243] (Fig. 1). PPARβ/δ regulates fatty acid catabolism in the muscle of monogastric animals [244]. Recent studies revealed this PPAR isotype playing essential roles in many more functions than regulation of lipid metabolism in the muscle, including FA oxidation in the liver and immune system [245]. There are few studies in ruminants about PPARβ/δ. It was originally hypothesized that this PPAR isotype plays a role in regulating glucose uptake and lactose synthesis in bovine mammary gland [4, 5] but *in vitro* data using bovine mammary cells did not support the hypothesis [246]. However, data indicated a role of PPARβ/δ in lipid metabolism in goat mammary cells, particularly FA oxidation and lipid secretion [247]. The PPARγ has been the most studied in dairy cows. In monogastrics this PPAR isotype is a pivotal player in adipogenesis and insulin sensitivity, but in dairy cows it has been specifically studied for its role in modulating milk fat synthesis [248–250], its involvement in the mTOR pathway, particularly mTORC2 [251], and its anti-inflammatory role [4, 5]. All PPAR isotypes play a role in improving reproductive parameters of dairy cows, such as the mitigation of endometritis [252], contribution to folliculogenesis [253], reproductive hormone synthesis [253], and the development of embryos [254]. All PPAR isotypes were found to be upregulated in response to naturally occurring prostaglandins in bovine uterine cells [255]. All above support a role of the three PPAR isotypes in bovine reproductive health.

In studies previously reviewed [4, 5], all PPAR isotypes, including PPARγ, appears to be activated mainly by SFA, with UFA only displaying partial or null activation. The insensitivity of PPAR to UFA, which is contrary to what observed in monogastrics [4, 5], is also supported by recent studies on the regulation of milk fat synthesis by *trans*-10,*cis*-12 CLA, which is known to depress milk fat synthesis [256]. PPARγ and some of its putative target genes were downregulated upon *trans*-10,*cis*-12 CLA supplementation in goat mammary cells and mouse mammary tissue [257, 258]. Though *in vitro* the PPARγ agonist rosiglitazone upregulates the transcription of several lipogenic genes in bovine mammary cells [72] and appears to be a central player in the mouse mammary response to CLA [258], supplementation of rosiglitazone *in vivo* did not attenuate milk fat depression caused by CLA in mice [259]. Similarly, the use of the putative PPARγ agonist 2,4-thiazolidinedione did not prevent milk fat depression induced by CLA in dairy sheep [260]. Data from our laboratory do not support 2,4-thiazolidinedione be a PPARγ agonist [4, 261, 262]. As argued previously [4, 263], PPARγ is one of the players in a larger network of transcription factors regulating milk fat synthesis, with a pivotal role of SREBP1. The presence and activation of SREBP1 is essential for milk fat synthesis and PPARγ appears unable to rescue milk fat synthesis once SREBP1 activity is compromised.

The study of PPAR activation by FA was mainly carried out using transcription of established or putative target genes based on studies in monogastric species. As argued previously [4], there is the need to use more precise molecular techniques to study the activation of each PPAR isotypes by FA. Using a combination of a PPARγ synthetic agonist and inhibitor with various LCFA in goat mammary cells, it was determined that C18:0 is an agonist of PPARγ but not C16:0. Interestingly, in that study it was observed that a basal activation of PPARγ is essential for the down-regulation of lipogenic genes by CLA, while activation of PPARγ blocks the negative effect of DHA on the same genes [195]. More recent data generated using a gene reporter assay support C16:0 to be an agonist of PPARα and PPARβ/δ but not PPARγ in bovine mammary and hepatic cells [168].

Evidences for activation of ruminant PPAR *in vivo* exist: activation of PPARα with a synthetic agonist increased hepatic FA oxidation in goats [264] and beef steers fed a whole shelled corn diet, higher in fat, had greater transcription of PPARα and PPAR target genes [265]; however, supplementation of FA *in vivo* do not always activate PPAR [4]. Additionally, PPARβ/δ and PPARγ, but not PPARα are activated by circulating NEFA present in plasma of early lactation cows [168]. Finally, an interesting but yet unexplored avenue is the activation of bovine PPAR by non-FA ligands, such as those naturally occurring in plants, such as boiogito [266], honokiol [267], berberine [268], and cannabinoids [269].

### Sterol regulatory element-binding proteins

The SREBP exist in three isoforms: SREBP1a, SREBP1c, and SREBP2. The SREBP are part of the transcriptional regulators of the basic helix-loop-helix leucine zipper family. The mechanism of SREBP formation and activation is well established as previously reviewed [157]. The immature and inactive SREBP is retained in the ER by the interaction with the cleavage-activating protein (SCAP) and insulin induced gene 1 (INSIG1). The latter retains the complex in the ER preventing SREBP activation. Upon lower level of cholesterol or FA, the INSIG1 is degraded after ubiquitylation and releases the SCAP-SREBP complex. SREBP is then cleaved and the N-terminal domain relocates to the nucleus and activates transcription of SREBP target genes via a sterol-regulatory elements in the promoter region of target genes [157]. SREBP are involved in the regulation of lipid metabolism, with SREBP1 mainly involved in FA synthesis and desaturation (especially SREBP1c), and SREBP2 in cholesterol biosynthesis [270].

Research on the role of SREBP in ruminant was initiated with the work of Bauman's group at Cornell in the regulation of milk fat synthesis, specifically in milk fat depression, as previously reviewed [263]. More evidences have accumulated providing a strong support for this transcription factor to be a central player in the control of milk fat synthesis in several ruminant species [271–275]. Its regulation of milk fat appears to be by a direct regulation of milk-fat synthesis related genes and through mTOR, as previously proposed [263]. Interestingly, it has been posited that the connection between SREBP1 and the mTOR pathway is through regulation of nuclear entry of lipin 1 (phosphorylated by mTOR), which would inhibit activity of SREBP1 [276]. Interestingly, *LPIN1* is a target of PPAR, which would suggest a possible connection between PPAR and SREBP1 as well [5].

Unlike other receptors, SREBP do not bind FA directly; however, the effect of FA on SREBP is indirect [263]. Activity of SREBP1 is inhibited by UFA, particularly PUFA [157]. The UFA can interact with ubiquitin regulatory X domain-containing protein 8, an essential enzyme for the ubiquitylation of INSIG1, inhibiting its action and, thus, reducing the activity of SREBP, as demonstrate in monogastric species [157]. Besides the role of UFA on SREBP, also SFA can affect this TF. In an *in vitro* study in bovine mammary epithelial cells, upregulation of SREBP1 and its target genes was detected in response to C18:0 treatment [271]. In the same study, treatment with C18:0 also increased the transcription of *PPARG*. It is possible that the effect of C18:0 is not directly on SREBP1 but via activation of PPARγ, as previously argued [263]; however, the relation between SREBP1 and PPAR in bovine mammary and the concerted regulation of milk fat synthesis is far from being fully elucidated.

Additional nutritional compounds beside FA can regulate SREBP1 in bovine mammary epithelial cells, such as methionine [277] which is partly mediated by FABP5 [278]. Alongside milk fat synthesis, SREBP1 plays  other roles in dairy cows, such as the demonstrated role in regulating homeostasis of the sodium/iodide symporter in mammary epithelial cells, as observed in humans [279], and it is involved in liver lipid metabolism in peripartum cows [263], including regulation of TAG synthesis in bovine hepatocytes [280, 281]. Interestingly, the mRNA abundance of *SREBF1* and *SREBF2* is remarkable similar between jejunum, liver, and mammary tissue in dairy cows (Fig. 1). Based on these results, dietary modulation of SREBP in ruminants would seem an avenue worth investigating.

### Hepatocyte nuclear factor 4

A member of the nuclear hormone receptor superfamily, the hepatocyte nuclear factor 4 (HNF4) is a transcription factor mainly involved in the regulation of transcripts in the liver [282]. Its canonical activation mechanism is different than that of PPAR: while PPAR form a heterodimer with RXR, HNF4 bind its response element as a homodimer, that is, a fusion of two HNF4 monomers [283]. Two isotypes of HNF4 (HNF4α and HNF4γ) have been characterized. Among the two, HNF4α is the most important for the liver and intestine. In dairy cows, the transcription of the two HNF4 is very low in mammary tissue but more abundant and similar in intestine and liver (Fig. 1). In adult mice, HNF4α is involved in the regulation of inflammation in the liver and intestine [284], mediating cytokines relays in hepatocytes and tight junction formation in the intestine [285], and it is associated with transcriptional regulation at the early stages of embryonic hepatocyte differentiation [286], possibly through remodeling of chromatin structure and the ensuing modulation of transcripts [287]. In the intestine HNF4α regulates FA oxidation and differentiation, as recently observed in mice [288].

In ruminants, information on the role of HNF4 is scarce. Possible roles of this TF in ruminants were previously reviewed [4, 263]. Very little information on the function of HNF4α was generated after those reviews. A recent study has found HNF4 binding sites in the proximal promoter of the cytochrome P450 3A gene in the bovine genome, suggesting that HNF4 might play a role in the response to xenobiotic [289]. While a complete picture of the ligands of HNF4 is not available, evidence in monogastrics supports a role of FA and acyl-CoA thioesters in the regulation of its activity: HNF4α was found to bind strongly to linoleic acid [290], as well as C17:0 cyclo, and, to a less extent, C16:0 [291]. Whether HNF4 can be activated by dietary FA or other dietary compounds in dairy cows remains to be determined.

### Liver X receptor

Discovered and subsequently characterized in 1994 [292], LXR is another member of the nuclear receptor superfamily. Two LXR isotypes are known, LXRα and LXRβ (coded by *NR1H3* and *NR1H2*, respectively). Transcription of LXR isoforms is somewhat similar between liver, intestine, and mammary tissue, with a higher mRNA abundance of *NR1H3* in liver and intestine compared to mammary tissue (Fig. 1). Both LXR isoforms play a crucial role in regulating cholesterol homeostasis [293]. As such, it is perhaps unsurprising that the main ligands of LXR are oxysterols, monooxygenated derivates of cholesterol [294]; other known LXR ligands are *D*-glucose and *D*-glucose-6-phosphate [295]. LXR activation is achieved through the formation of a heterodimer with RXR, and the subsequent binding to an LXR response element in the promoter region of target genes [296].

In monogastrics LXR is involved with regulating cholesterol excretion in the intestine [297], generating more cholesterol by shifting acetyl-CoA from cholesterol biosynthesis to *de novo* FA synthesis, mainly via regulation of SREBP-1c [298] and fatty acid synthase (FASN) [299], and regulating intestinal absorption of cholesterol [300].

Research on the role and possible dietary activation of LXR in ruminants is still in its infancy and was previously reviewed [4, 263]. More recent data support important role of both LXR isoforms in regulating milk fat synthesis partly via modulation of *SREBF1* transcription in goat mammary epithelial cells [273, 301, 302]. In bovine vascular cells activation of both LXR isoforms increased synthesis of SFA, particularly C18:0, and MUFA and increased calcification of the vascular cells [303]. In the same study, it was demonstrated that C18:0 increases mineralization of the cells by increasing transcription of alkaline phosphatase. Unfortunately, the researchers did not test if C18:0 is an agonist of LXR, but it might be a possibility. A role of the LXR pathway in determining milk cholesterol content was postulated by using data from a genome-wide association study [304]. Data in monogastrics indicate a negative effect of UFA on the activity of LXR [225]; however, the effect of UFA is not fully substantiated as previously argued [4]. To our knowledge, there are not data available in ruminants on the role of FA in modulating LXR.

### Free fatty acid receptors

Besides TF, also the G protein-coupled receptors (a.k.a., FFAR) can sense the level of extracellular FA and affect the biology of cells, although not directly by altering the transcription of specific genes. The FFAR affect metabolic functions, including glucose metabolism, and regulate immune system, especially FFAR2 and FFAR3, that are activated by SCFA, as recently reviewed for monogastrics [305, 306].

An excellent review on functions and activation by FA of FFAR in dairy cows was recently published [307]. According to that review, FFAR1 and FFAR4 are activated by LCFA, especially UFA, and FFAR2 and FFAR3 are activated by SCFA. All play important roles in the innate immune system with an apparent pro-inflammatory role, especially FFAR1, involving neutrophils and mammary epithelial cells. Recently, it was demonstrated a role of DHA in activating FFAR4 in bovine neutrophils increasing superoxide production which could improve the killing capacity of those cells [308]. The FFAR also play a role in reproduction in dairy cows, as in bovine endometrial cells and granulosa cells DHA activates FFAR4 inducing intracellular release of calcium in the endometrium [309], important for prostaglandins synthesis, and proliferation of the granulosa cells [310]. The FFAR1 and the FFAR2 were detected to be expressed in liver of peripartum cows with the transcription of FFAR1 being decreased by high level of BHBA, although the roles of those receptors in the liver of dairy cows is still unclear [311] and their transcription is relatively low in liver of dairy cows (Fig. 1).

## Role of dietary fatty acids on milk FA composition and health and performance of dairy cows

Dietary FA affect milk fat but also affect reproductive performance and can help animals to cope with the biological stress during the peripartum. In this section, we review the most recent findings associated with the role of dietary FA on milk fat composition, especially the ones associated with human health, and their role in health and performance of dairy cows.

Milk is an interesting food matrix where fat is present mostly as TAG (98%), and other type of lipids such as DAG (around 2% of the lipid fraction), cholesterol (less than 0.5%), phospholipids (about 1%) and free FA (about 0.1%) [312] are a minor part. Milk FA are originated from two sources, the preformed FA found in the feed and from microbial activity in the rumen [313]. In other words, FA in bovine milk are produced by *de novo* synthesis in the mammary gland mostly using byproducts of the rumen microbial fermentation or taken up from plasma lipids. The latter are also somewhat a mixture of dietary FA, microbial FA, and *de novo* synthesized FA mainly produced by the adipose tissue also using acetate from the rumen. Generally, C4:0 to C14:0 and some C16:0 are produced *de novo *in the mammary gland [314]. Preformed FA are generally considered to contain approx. half of the C16:0 and all the other FA with more than 16 carbon atoms [315]. Most of the FA in milk are SFA (such as C16:0 and C18:0) with high amount of some UFA (such as *cis-*9 C18:1) (Table 1).

**Table 1:** Typical fatty acid profile from retail whole milk in Chile (3.5 % of fat content)

**Fatty acids, g/L**	**Mean ± SE**
*De novo* synthesis	
C4:0	1.18±0.18
C6:0	0.68±0.01
C8:0	0.37±0.01
C10:0	0.87±0.03
C11:0	0.10±0.01
C12:0	1.04±0.04
C13:0	0.02±0.001
C14:0	3.62±0.14
C14:1 *cis*-9	0.17±0.06
C15:0	0.34±0.02
C15:1 *iso*	0.07±0.004
*De novo* synthesis and performed	
C16:0	9.89±0.29
Preformed fatty acid	
C16:1 *cis*-9	0.48±0.05
C17:0	0.23±0.04
C17:1 *cis*-9	0.07±0.01
C18:0	2.84±0.40
C18:1 *trans*-9	0.01±0.01
C18:1 *trans*-10	0.007±0.01
C18:1 *trans*-11	0.22±0.09
C18:1 *cis*-9^a^	6.23±0.41
C18:2 *trans*-9, *trans*-12	0.09±0.01
C18:2 *cis*-9, *cis*-12	1.31±0.74
C20:0	0.40±0.29
C18:3 *cis*-6, *cis*-9, *cis*-12	0.03±0.01
C18:3 *cis*-9, *cis*-12, *cis*-15	0.05±0.03
C18:2 *cis*-9, *trans*-11	0.05±0.03
C21:0	0.20±0.05
C20:2	0.009±0.002
C20:3 n-6	0.03±0.02
C20:3 n-3	0.03±0.01
C20:4 n-6	0.02±0.005
C22:2	0.013±0.003
C20:5 n-3	0.01±0.004
C22:6 n-3	0.03±0.01
Σ Saturated fatty acids	21.85±0.30
Σ Monounsaturated fatty acids	7.28±0.50
Σ Polyunsaturated fatty acids	1.73±0.71

Adapted from Vargas-Bello-Pérez et al [316]. Data was based on 8 samples

^a^18:1 *cis*-9 is partly derived from the delta-9 desaturase activity, which can be considered part of *de novo* synthesis, although preformed FA are used as substrate

### Role of dietary fatty acids on milk yield

The response of dairy cows in term of milk yield to fat supplement is likely mostly the consequence of the energy content of the fat as it provides energy to sustain lactation and other energy expenditures in productive cows. Because of this and the fact that early post-partum cows experience negative energy balance, fat supplementation could have greater effects during early lactation than in late lactation [317].

Milk yield is mostly driven by dry matter intake. Supplementing dairy cow diets with high amounts of UFA often cause a drop in feed intake, as discussed in the “Inhibition of feed intake by dietary FA” section of this review. The drop in DMI by high amount of dietary UFA can negatively affect milk yield [318]. However, as observed in the various studies presented in Table 2, when animals are fed with free oils (i.e., soybean and linseed oils) at an inclusion of ≤3% DM, there is not a detrimental effect on milk yield and may be associated to the fact that at that inclusion rate these oils do not affect DMI [154]. Bu et al. [332], supplemented cows with soybean oil or fish oil alone or in combination at 4% DM resulting in an increase in milk yield compared with the control treatment with no added fat. Other experiments with higher amounts of oil supplementation have not reported negative effects on milk yield such as Huang et al. [333] where animals were fed with either 5% DM of soybean oil, 5% DM of Ca salts of CLA or 5% DM of both. Similarly, in a grazing system, cows were fed with linseed oil at 2.5% DM, 5.1% DM and 7.7% DM without changes in milk yield [334].

**Table 2:** Effect of dietary vegetable or marine supplements on the fatty acid composition of bovine milk when animals are fed on corn silage-based diets

**Lipid supplements used**	**Effect on milk fatty acids**	**Milk yield**	**Ref.**
1) Control (corn silage based TMR) 2) Fish oil (FO; 3%DM) 3) Soybean oil (SO; 3%DM)	↑ SFA FO and SO ↑ *trans-* C18:1; ↑ *cis-*9, *trans-*11 C18:2 by FO	No effect	[319]
1) Control (corn silage based TMR) 2) Olive oil (OO; 3%DM) 3) Hydrogenated palm oil (HPO;3%DM)	↓ SFA by OO ↑ *cis-*9 C18:1; ↑ *cis-*9, *cis-*12, *cis-*15 C18:3 by OO	↑ by OO	[320]
1) Control (corn silage based TMR) 2) Soybean oil (SO; 3%DM) 3) Hydrogenated palm oil (HPO; 3%DM)	↑ C18:1 *trans* including *trans-*11 C18:1 by SO	No effect	[321]
1) Control (corn silage based TMR) 2) Fish oil (FO; 2.6%DM) 3) FO (1.3%DM) + HPO (1.3%DM)	↑ C18:1 *trans*-11; ↑ DHA; ↓ C6:0, C8:0, C10:0 and C14:0 by FO	No effect	[322]
1) Control (corn silage based TMR) 2) Soybean oil (SO; 2.3%DM) 3) Linseed oil (LO; 2.3%DM)	↑ PUFA; ↑ *cis-*9, *trans-*11 C18:2; ↓ SFA by SO and LO	No effect	[154]
1) Control (corn silage based TMR) 2) Extruded linseed (EL; 22 g/kg DM) 3) Ca salts of palm and linseed oils (PLO; 22 g/kg DM) 4) Milled rapeseed (MR; 22 g/kg DM)	↑ CLA; ↓ SFA by EL, PLO, and MR	↑ by EL ↑ by PLO ↑ by MR	[323]
1) Prilled fat (3.5%DM) Unprocessed oilseeds: 2) Rapeseed (6.9%DM) 3) Cottonseed (18.4%DM) 4) Linseed (7.5%DM)	↑ MUFA; ↑ PUFA by all three unprocessed oilseeds	No effect	[324]
1) Control (corn silage based TMR) 2) Microalgae 2 g/kg DM 3) Microalgae 4 g/kg DM 4) Microalgae 6 g/kg DM	↓ C18:0, ↓ *cis-*9 C18:1, ↓ *cis-*9, *cis-*12 C18:2; ↓ *cis-*9,12,15 C18:3; ↑ *cis-*9,*trans-*11 C18:2; ↑ *trans-*9 C18:1; ↑ *trans-*11 C18:1 by microalgae	No effect	[325]
1) Control (corn silage based TMR 2) Rubber seed oil (RO; 4%DM) 3) Flaxseed oil (FO; 4%DM) 4)2%DM of RO and 2%DM of FO	↑ *trans-*11 C18:1; ↑ *cis-*9, *trans-*11 C18:2 by RO and FO	↑ by RO ↑ by FO ↑ by RO + FO	[326]
1) Control (corn silage TMR) 2) Palmitic acid TAG (PA; 1.7%DM) 3)Ca-salts of palm FA (CAF; 1.8%DM)	↓ *de novo*/preformed FA by PA and CAF ↑ C16:0 by PA	↑ by PA and CAF	[327]
1) Control (sorghum silage TMR) 2) Palmitic acid (PA; 4%DM)	↑ SFA by PA	↑ by PA	[152]
Corn silage TMR’s with: 1) Ca salts of palm oil (PA; 12.3%DM) 2) Corn grain and wheat (GW; 13.4% corn silage, 4%DM barley grain, 12%DM wheat starch, 25%DM wheat middlings) 3) Extruded rapeseeds (RS; 24.5%DM) 4) Extruded sunflower seeds (SS; 24.5%DM)	↑ SFA by PA and GW ↑ MUFA by RS and SS	No effect	[328]
1) Corn silage TMR 2)C16:0 (PA; 1.5%DM) 3)C16:0 and C18:0 (MIX; 1.5%DM)	↓ *de novo* synthesis by PA and MIX ↑ C16:0; ↑ *cis-*9 C16:1 by PA and MIX	No effect	[329]
1)Control 2)C16:0 (PA; 2%DM) 3) Ca salt of C16:0 (CaPA; 2%DM)	↓ *de novo* synthesis by PA and CaPA	No effect	[330]
1) Enriched C16:0 (P; 20 g/kg DM) 2)C16 + C18 (PS; 13 and 7 g/kg DM) 3)C16 + C18 (SP; 7 and 13 g/kg DM) 4) Enriched C18:0 (S; 20 g/kg DM)	↑ UFA by S ↑ *de novo* FA by S	No effect	[331]

↑ = increase; ↓ = decrease.

The forage to concentrate ratio (F:C) can play an important role in determining the effect of supplemented oils on milk yield in dairy cows [1]. As an example, in the study by Ueda et al. [335], supplementation of up to 3% DM of linseed oil depressed rumen digestibility only in dairy cows receiving a low F:C ratio (35:65) but not in cows receiving a high F:C ratio (65:35). Jenkins and Harvatine [336] argued that a proper fat supplementation to dairy cows should account for the total UFA present in the fat supplemented and the amount of dietary fiber. Therefore, it is crucial to balance the amount of fat supplemented in the diet, which should not exceed 5% DM, and the amount, type, and the digestibility of dietary fiber [337]. Other factors affecting DMI when animals are supplemented with oils are palatability, chain length and saturation of FA, and the form of fat (e.g., free FA, TAG, glycolipids) [338].

If all the above variables are considered, then the animal should be able to benefit from the energy supplied by the dietary FA and have a proper rumen function. Taken together, different effects of oil supplements on the DMI and milk yields presented in Table 2 may be attributed to the oil palatability, the amounts added, and the varying F:C ratio [326].

In Table 2 are summarized six recent studies were dairy cows were supplemented with SFA. In two out of six studies milk yield was increased in animals supplemented with SFA (all using C16:0) with not effects of SFA supplementation on milk yield in the other four studies, Thus, feeding SFA can improve milk yield but not always.

### Role of dietary fatty acids on milk fat composition

Today, there is an active debate on whether consuming milk fat is positive for human health [339]. It is known that unique individual milk FA are bioactive and can prevent metabolic diseases at least in animal models [340]. Nutrition of dairy cows is the most practical and economical way to increase the contents of bioactive FA in milk and dairy products. Efforts have been done to modulate the milk FA profile towards a healthier fat (less SFA) for human consumption. A metanalysis on the effect of supplementing oil to milk fatty acid composition in dairy cows was published more than 10 years ago [341]. Since then, extensive research was accomplished in assessing the supplementation of different feedstuffs to improve milk fat composition such as microalgae, cottonseed, flaxseed, extruded soybean, extruded linseeds, rubber oil, fish oil, soybean oil, hydrogenated vegetable oil, calcium salts of palm and fish oil (Table 2).

Most of the studies presented in Table 2 encompass the use of UFA to supplement dairy cows. These studies were designed to reduce the total contents of SFA in milk [319, 320, 323]. Cows were fed with different lipid sources, most of them UFA that promoted a shifts in the rumen biohydrogenation process and thereby increasing intermediate byproducts (i.e., CLA and *trans-*11 C18:1), and decreasing SFA (i.e., C16:0 and C18:0). Marine feedstuffs such as fish oil and microalgae have strong impacts on rumen biohydrogenation that often leads to increase in some *trans* C18:1 and CLA [322, 325]. Dietary vegetable oils such as soybean oil often leads to changes in ruminal microbial populations and shifts in ruminal fermentation parameters affecting cellulolytic bacteria such as *Fibrobacter succinogenes, Ruminococcus flavefaciens, Ruminococcus albus* and *Butyrivibrio fibrisolvens* that are important in the biohydrogenation process of PUFA [342]. Cellulolytic bacteria are affected by dietary supplementation of lipids with high concentrations of PUFA, as discussed in the “Effects of fatty acids on microbiota of the rumen” section of this review.

Production systems can also be different in their milk FA profile, for example, compared with cows fed on total mixed ratios (based on corn silage), milk of cows fed on pasture-based diets (either full grazing or based on grass silage) has higher concentration of some UFA such as various *trans* C18:1, C18:2, and C18:3 [313]. The high proportion of C18:3 in milk of grazing cows is due to the high amount of this FA in the galactosyl diacylglycerols constituting the thylakoids [343], the main membrane structure of the chloroplast. Due to the large amount of PUFA and the concomitant high fermentability, fresh pasture can induce milk fat depression [344]. However, the rumen dynamic of biohydrogenation of PUFA in fresh grazing plants is complex and is affected by several factors, as reviewer by Buccioni et al. [343]. The biohydrogenation of C18:3 in the rumen is decreased when this FA is associated with the membrane fraction [343]. Recent *in vitro* data demonstrated that biohydrogenation of PUFA is decreased when associated with complex lipid fractions, such as phospholipids and cholesterol ester, compared to simple lipid fractions, such as TAG [17]. The utilization of FA from grazing plants is also determined by the composition of the pastures. There are secondary compounds that can affect the lipolysis and rumen utilization of plant-derived FA in the rumen, such as the presence of polyphenol oxidase in red clover that protect glycerol-based lipid from lipolysis in the rumen [345].

### Effect of dietary fatty acids on reproductive performance

Dietary FA are crucial in the reproductive performance of dairy cows due to their influence on the energy balance and reproductive processes that are not just related to energy supply [346]. Fat supplementation has been associated with positive and negative effects on reproduction [347] and the amount of supplemental fat needed to elicit an effect on reproductive function may vary. For example, while some studies indicated that the amount of added vegetable oil necessary to maximize positive ovarian effects is not less than 4% DM [348], others have reported that fat supplementation at 3% DM has often positive influence in the reproductive status of the dairy cows [349].

Feeding fat to cattle generally improves establishment and maintenance of pregnancy. Potential improvements in fertility of cows caused by supplementing cows with fat have generally been associated with enhanced follicle development postpartum, increased diameter of the ovulatory follicle [346], increased progesterone (PG) concentrations during the luteal phase of the cycle [349], altered uterine/embryo cross-talk by modulating PG synthesis, and improved oocyte and embryo quality [350]. Some of these effects have been more influenced by the type of FA than by fat feeding per se as differential responses *in vivo* to FA feeding suggest that UFA of the n-6 and n-3 families are most beneficial for fertility [351, 352].

#### Unsaturated fatty acids

Lipids play critical roles in the structure and function of cell membranes and cytoplasm of oocytes affecting their development competence [353]. Diets enriched with n3 PUFA increase membrane fluidity and n-3 PUFA derived eicosanoids, such as prostaglandins, leukotrienes, thromboxane and lipoxins [354]. In monogastrics, the n-3 PUFA bind FFAR1 and FFAR4 [306] affecting fertility as FFAR4 can modulate signaling of mitogen-activated protein kinases MAPK1/3 and MAPK14, which are known to be involved in bovine granulosa cell proliferation and steroidogenesis [310]. The FFAR1 and FFAR2 are expressed in the endometrium and are activated by DHA inducing increase in intracellular calcium mobilization, important for uterine contraction and prostaglandin synthesis [309].

Addition of C20:5n-3 to granulosa cells *in vitro* increases progesterone and estradiol secretion with concomitant increase in protein expression levels of several steroidogenic enzymes and the cholesterol transporter steroidogenic acute regulatory protein (a.k.a. StAR) [310]. Increase in progesterone level was also reported in sheep after feeding n-3 enriched diet [355]. These effects on granulosa cell function could thus be related to the improved reproduction observed after n-3 enriched diet supplementation.

Compared to dietary supplementation with SFA where palmitic acid is predominant, dietary PUFA from rumen-protected fish oil improve pregnancy and decrease pregnancy loss in dairy cows [356]. Similarly, when cows are supplemented with 10 g of C20:5n-3, their rate of pregnancy and pregnancy per artificial insemination are increased [357]. Enrichment of *n-3* PUFA in membrane of ovarian compartment can also affect prostaglandins synthesis in cows as demonstrated by supplementation of dairy cows with extruded linseed [358].

Besides affecting membrane composition of reproductive cells, the positive effect of FA on fertility can be exerted via PPAR as mentioned in the section “Peroxisome proliferator-activated receptors” in this review. All three PPAR isotypes are important for early embryo development, fetus development, and in various compartments of the reproductive system (i.e., uterus and testis) of many species, including cattle [359]. However, PPARγ appears to be the most important in monogastrics and is associated with ovarian function and female fertility [360], but it is also the most studied in ruminants fertility. PPARγ is expressed and activated during all the stages of bovine embryo development (both in the inner mass and in the trophectoderm) and in the placenta (cotyledons and caruncles) in bovine and sheep [5]. From the review of the literature by Ribeiro [361], PPARγ appears to coordinate lipid metabolism of trophectoderm cells which is crucial for conceptus elongation and survival. Ribeiro suggested a role of PUFA, and their derivatives, present in the histotroph as important natural ligands of PPARγ in the trophectoderm cells affecting conceptus biology. This is a very interesting idea with tremendous practical application; however, fundamental data are still needed since activation of bovine PPARγ by PUFA has not yet been demonstrated. Furthermore, data obtained from mammary, liver, and kidney cells from ruminants indicate a minor effect of UFA and PUFA on activation of PPAR [4, 5].

#### Saturated fatty acids

The agonistic effect of SFA on bovine PPAR [4] could suggest a positive effect on reproduction. Saturated FA are instead detrimental to oocyte and embryo development compared to UFA [362]. In fact, oleic acid in cumulus cells protects oocytes from lipotoxic effects from C16:0 or C18:0 [363]. Feeding fats enriched with UFA at the beginning of the dry period and during the postpartum period improves postpartum reproductive health and milk production [1]. Silvestre et al. [356] fed cows with Ca salts of safflower oil (1.5% DM) from 30 d prepartum until 30 d postpartum and then Ca salts of fish oil (1.5% DM) from 30 to 160 d postpartum. Overall, pregnancy rate was greater in cows fed Ca salts of unsaturated lipid sources compared to animals that received Ca salts of palm oil. This feeding management also aided to improve embryo development and subsequent pregnancy rates [1]. The negative role of SFA on reproduction is not fully elucidated. It is possible that SFA induce increase in systemic oxidative stress [145] or cellular ER stress damaging reproduction [364]. Induction of ER stress by SFA was observed in liver of monogastrics [365]. Not similar data are available for dairy cows; however, a mixture of SFA and UFA typical of NEFA in early post-partum elicited a dose-dependent increase in ER stress in calf hepatocytes [366].

#### Role of unsaturated fatty acids on prostaglandin F_2α_ metabolite

The influence of n-3 and n-6 PUFA on circulating PG concentrations have been variable due to the type of lipid and dietary supplements fed [351], parity and days post-partum [367]. Prostaglandin F_2α_ metabolite (PGFM) concentrations following stimulation are a measure of the potential of the animal to secrete prostaglandin in response to a stimulus. Prostaglandin is commonly stimulated by an injection of oxytocin, as this is indicative of PG release in response to endogenous oxytocin [368]. Interestingly, as summarized in Table 3, the results are variable between studies; however, the overall effects appear to be consistent, with n-6 FA (for example from soybeans byproducts) increasing and n-3 FA (for example from linseed and fishmeal) decreasing PGFM response to oxytocin. Few studies reported a lack of any effect of PGFM response to fat supplement [372, 373]. The reason for such an effect is not clear; however it might be due to the small difference in n-6:n-3 ratio between diets [368] and this factor needs to be accounted for improving reproductive responses when animals are fed with PUFA.

**Table 3:** Effect of dietary polyunsaturated fatty acids on oxytocin-stimulated prostaglandin F_2α_ metabolite (PGFM) in dairy cows

**Animals**	**Treatments**	**Oxytocin**	**Results**	**Ref.**
Holstein 116 DIM* Day 15 of synchronized oestrous cycle	1) 2.6% fish meal (FM) 2) 5.2% FM 3) 7.8% FM 4) Corn silage, corn grain, soybean meal	100 IU	PGFM response lower in cows fed FM	[369]
Holstein 25 DIM Day 15 of synchronized oestrous cycle	Maize silage, alfalfa, maize supplemented with: 1) Extruded linseed (5.5%) 2) Extruded soybean (4.9%)	100 IU	PGFM not significantly lower at base line with linseed. PGFM response was not different between treatments.	[370]
Holstein 38 DIM Day 14 of synchronized oestrous cycle	Grass and maize silage, barley supplemented with: 1) Flaxseed (4%) 2) Megalac (4%) 3) Sunflower seeds (4%)	20 IU	PGFM response higher with sunflower seeds and lower with flaxseed	[371]
Breed not described 38 DIM Day 14 of synchronized oestrous cycle	1) Corn silage, alfalfa hay, ground corn, soybean meal supplemented with: 2) 1.25% fish meal (FM) 3) 2.5% FM 4) 5.0% FM 5) 2.3% Ca salts of fish oil fatty acids	100 IU	PGFM response was not different between treatments.	[372]
Holstein 63 DIM Day 15 of synchronized oestrous cycle	Grass + maize silage, barley supplemented with: 1) Flaxseed (10%) 2) Megalac (4%) 3) Micronized soybeans (17%)	100 IU	PGFM response was not different between treatments.	[373]

*Day in milk

#### Unsaturated fatty acids and the reduction of negative energy balance

PUFA enriched diets in cattle are known to reduce the extent of negative energy balance (NEB) experienced in early lactation [374]. Supplementation of PUFA positively affects the oocyte metabolism [375]. For example, DHA was found to modulate lipid metabolism in oocyte-cumulus complex and improve oocyte cytoplasm maturation during *in vitro* maturation [376]. The size of dominant follicles is reported to increase in cows fed with PUFA diets as compared to cows fed with MUFA [351]. Increased follicle size improves oocyte quality and corpus luteum function in cows [377]. For example, larger ovulatory follicles with lower rates of pregnancy losses were found in cows fed with rolled flaxseed compared to those fed with rolled sunflower seeds [378].

#### Additional effects

Among other dietary FA, rumenic acid increases plasma concentration of insulin-like growth factor-1 (IGF-1) in cows and consequently promoting conception rates [379]. Recently, CLA was reported to affect follicular granulosa cells morphology and function, which may result in a compromised ovarian cyclicity and impaired fertility [380].

### Effect of dietary fat on health of transition cows

The transition from late gestation to early lactation is regarded as one of the most challenging period in the life of dairy cows [223]. Reasons for feeding fat during the transition period are several. The degree of lipid supplementation has been mostly within the range of traditional recommendations (normally 3 to 4% of supplemental lipid to a maximum of 6% of DM as total lipid) [381]. It is important to note that extreme rates of lipid mobilization lead to increased uptake of NEFA by liver and increased TAG accumulation in this tissue. Then, if this lipid infiltration becomes severe, the syndrome of hepatic lipidosis or fatty liver may result, which can lead to prolonged recovery and lastly, the animal can die [382]. Increased lipid accumulation and decreased glycogen in the liver are associated with an increased susceptibility to induction of ketosis [223].

As previously reviewed, supplementation of dairy cows early post-partum with SFA, especially palmitic and stearic acids are beneficial to dairy cows [3]; however, the effect of supplementing early post-partum cows with UFA is less clear. Increasing the energy supply by feeding dietary fat sources or decrease energy expenditure by supplying specific FA such as *trans-*10, *cis-*12 CLA to decrease milk fat output during early lactation, do not appear to be beneficial, since they fail to decrease the concentration of circulating NEFA early post-partum, the major index of NEB [379, 383, 384]. Bernal-Santos et al. [384] fed cows a mixture of CLA isomers as Ca-salts from 14 d prepartum through 140 d postpartum and reported that concentration of TAG in liver was not affected by feeding CLA. In addition, in that study, milk fat percentage and yield decreased during the first three weeks postpartum. Similar results were obtained by Castañeda-Gutiérrez et al. [379], who reported similar effects on milk fat percentage and yield beginning during the third week postpartum in response to feeding CLA. Selberg et al. [383] fed cows with calcium salts of CLA and trans-octadecenoic acids during the transition period and early lactation and reported that TAG in liver decreased in response to feeding the trans-octadecenoic acid source. Those studies dealing with CLA supplementation were based on the rationale that CLA should decrease energy output by decreasing milk fat yield during early lactation [385] and in turn it decreases the extent and duration of NEB [382].

In cows on a grazing system, Kay et al. [386] fed animals with only pasture and pasture supplemented with hydrogenated palm oil (540 g/d) or rumen inert CLA (600 g/d) from 27 d prepartum to 36 d post-partum. They reported no treatment differences in plasma glucose, insulin, leptin, or NEFA concentrations. This study used *trans-*10, *cis-*12 CLA isomer and authors attributed those results to the fact that the supplemented cows had similar net energy balance.

Other strategies have considered the use of oilseeds by-products fed during the transition period. Petit and Benchaar [387] supplemented transition dairy cow with either whole flaxseed, a commercial product containing mainly palmitic, oleic, and linoleic acids (Megalac), or micronized soybeans from 6 weeks before calving to 120 days after calving. The authors detected no treatments effect on either milk composition or milk yield but concentration of plasma NEFA was higher with cows fed with Megalac compared with whole flaxseed and micronized soybeans. Santschi et al. [388] fed transition dairy cows from 40 d before to 40 d after calving with extruded linseed (1.8 kg DM/d). They did not find negative effects on plasma metabolites such as glucose, TAG, BHBA and NEFA. Both studies were based on the fact that flaxseed and linseed are excellent sources of C18:3, one of the most beneficial FA for the liver because its addition to bovine hepatocytes resulted in decreased TAG concentrations and greater rates of gluconeogenesis compared with other LCFA [389]. Leiber et al. [390] fed cows with crushed linseed as a source of linolenic acid and sunflower seed as a source of linoleic acid and a mix of both from 7 weeks before to 6 weeks after parturition. They reported that plasma metabolites (TAG, leptin, glucose, insulin, IGF-1, NEFA and BHBA) were similar between treatments.

The immune system also is positively affected by feeding prepartum cows with oilseed by-products as shown by Lessard et al. [391] who fed animals with calcium salts of palm oil (Megalac), micronized soybeans (rich in linoleic acid), or whole flaxseed (rich in linoleic and linolenic acid) for six weeks before calving. They reported that flaxseed increased interferon-γ compared to the other treatments. This is partly explained by the PUFA influence on cellular communication and activation through the synthesis of PG, tumor necrosis factor-α, and interferon-γ, [392].

## Conclusion

In this review, we have provided the most up-to-date information available on utilization of dietary FA and their effects on performance and health of dairy cows. We did not cover all the possible biological effects of FA, such as the role of dietary FA on the lipidome and insulin sensitivity in dairy cows during the peripartum which were recently reviewed [145]. However, based on the literature reviewed we can make some concluding remarks and propose some prospective research for each main topic covered in the present review.

### Utilization of FA in the rumen

Our review of the literature highlighted few advances made in the last decade or so on the utilization of FA by the rumen microbiota, with an emphasis on biohydrogenation of PUFA. Microbiota in the rumen can have a substantial effect on dietary lipids affecting the type and form of FA reaching the intestine. The microbiota can be rather negatively affected by various FA, especially UFA, and biohydrogenation can be considered a way to decrease toxicity of PUFA towards bacteria. We expect important advances in the next decade or so on the interactions between FA and rumen microbiota due to the rapid increase in data and data analysis via high-throughput techniques and associated bioinformatics tools. The large emphasis on environmental issues associated with dairy cows, such as methane production and N leaching, are both dependent from the activity of rumen microbiota; thus, a better understanding of the interaction between FA and rumen microbiota can aid in formulation of more efficacious dietary supplementation of FA. Furthermore, the complex effects of rumen microbiota on dietary FA, including the synthesis of volatile fatty acids, provide an additional complexity in the application of nutrigenomics via feeding FA in dairy cows.

### Absorption and utilization of FA by dairy cows

Once the FA arrive into the intestine the digestion and absorption is very effective in ruminants and it is becoming more clear the molecular processes and the main molecular players involved; however, advances in knowledge of those were mostly on monogastric animals. Thus, more research should be performed to understand the absorption of FA in dairy cows. Very important in this contest is the understanding of the nutrigenomic roles of various FA in the intestine, especially considering that several of the main transporters of FA are likely regulated by FA via PPAR. Thus, future studies should focus on dissecting the molecular mechanisms of FA absorption in ruminants and their nutrigenomic modulation.

Transport of FA from the intestine to peripheral tissue happens mainly via lipoproteins; however, most of the studies conducted in bovine date back few decades and there is a paucity of new data. Even less data are available for uptake of FA by cells, including liver. As for intestinal absorption of FA, major scientific advances were made in monogastric animals. This clearly indicate that more studies should be carried out in dairy cows to understand the molecular player involved in those processes.

### Nutrigenomic role of FA via activation of transcription factors

Among the few TF known to be modulated by FA, data support the activation of PPAR by SFA and inhibition of SREBP1 by PUFA in dairy cows. The roles of those TF are somewhat clear in bovine mammary tissue, with SREBP1, LXR isoforms, and PPARγ involved in the regulation of milk fat synthesis, but the role of the other TF or of all the above TF in other tissues remains somewhat unclear. Paucity of data exist on the activation of LXR isoforms and HNF4A by FA, especially in dairy cows. New data are being generated in the activation of bovine PPAR isotypes by FA, but more data are needed, such as the identification of FA that are agonists of PPAR and the dose of each of FA (or the combination of FA) that maximizes PPAR activation. The above information is important to move toward nutrigenomic applications via precision feeding.

Despite the above, it is clear that feeding dairy cows to tailor specific TF with FA (i.e., nutrigenomics) can be further complicated by the dynamic of FA released via LPL, that are only partly taken up by the cells, and the presence of circulating NEFA, also those partly derived from the LPL activity. The complexity of such dynamism makes *in vivo* prediction of FA dose that modulate a TF based on *in vitro* data difficult, indicating the need of complex *in vivo* experiments, such as dose-effect trials in combination with the use of more holistic approaches using techniques for high-throughput data in association with systems biology approaches [139].

### Effect of feeding dairy cows with supplemental fatty acids

Feeding FA to dairy cows can be very beneficial, especially considering the positive role of UFA for fertility and the enrichment of n-3 PUFA in milk. Despite great advances in techniques to protect FA from the rumen microbiota, rumen biohydrogenation remains an important challenge to fully exploit those beneficial effects. Furthermore, the reason for the beneficial effects of n-3 PUFA on fertility is not yet fully revealed. A positive effect of supplementing FA on milk yield is observed, but it is inconsistent. This is partly due to the complexity of the various processes FA undergo before reaching the various tissues, including the anorexic effect of UFA, but it is also due to a lack of knowledge on nutrigenomic effects of various FA in each tissue.

The development of Omic technologies as well as advanced molecular biology techniques to study ruminant nutrition has helped to improve our understanding of lipid biology. The complexity of the absorption and peripheral utilization of FA as reviewed above, the lack of specific molecular studies carried out in dairy cows about some of those processes, and the relatively poor understanding about TF that can be modulated by FA are all “black-boxes” that still need to be open considering that most of the advances of the molecular aspects of dietary FA metabolism were generated in monogastrics, which are different than ruminants.

## Data Availability

The datasets used and/or analyzed during the current study are publicly available.

## Abbreviations

ABC: ATP-binding cassette
ACSL: Acyl-CoA synthetase long chain
AGPAT: 1-acylglycerol-3-phosphate acyltransferase
ANGPTL4: Angiopoietin-like 4
apo: Apolipoprotein
APOBEC: APOB mRNA editing complex
BHBA: Beta-hydroxybutyrate
CACT: Carnitine acylcarnitine translocase
CLA: Conjugated linoleic acid(s)
CPT1: Carnitine palmitoyltransferase I
DAG: Diacylglycerol
DBI: Diazepam binding inhibitor
DHA: Docosahexaenoic acid
DGAT: Diglyceride acyltransferase
DIM: Day in milk
DM: Dry matter
DMI: Dry matter intake
ER: Endoplasmic reticulum
FA: Fatty acid(s)
FABP: Fatty acid-binding protein
FABPpm: Plasma membrane fatty acid-binding protein
FATP: Fatty acid transport protein
FFAR: Free fatty acid receptor
F:C: Forage to concentrate ratio
G3P: Glycerol-3-phosphate
GPAT: Glycerol-3-phosphate acyltransferase
GPIHBP1: Glycosylphosphatidylinositol-anchored high-density lipoprotein binding protein 1
HDL: High-density lipoproteins
HSL: Hormone-sensitive lipase
LCFA: Long-chain fatty acid(s)
LDL: Low density lipoproteins
LDLR: Low density lipoproteins receptor
LPIN: Lipin
LPL: Lipoprotein lipase
LXR: Liver X receptor
MCFA: Medium-chain fatty acid(s)
MCT: Proton-linked monocarboxylate transporters
MTTP: Microsomal triglyceride transfer protein
NEB: Negative energy balance
NEFA: Non-esterified fatty acids
NPC1L1: Cannalicular sterol transporter Niemann-Pick C1-like 1
PG: Progesterone
PGFM: Prostaglandin F2α metabolite
PPAR: Peroxisome proliferator-activated receptor
PUFA: Poly-unsaturated fatty acid(s)
RXR: Retinoid X receptor
SCFA: Short-chain fatty acids
SFA: Saturated fatty acid(s)
SLC27A: Solute carrier family 27
SR-B1 or SCARB1: Scavenger receptor class B type 1
SMCT: Sodium-coupled monocarboxylate-transporter
SLC9A: Sodium-coupled monocarboxylate-transporter
SREBF: Sterol regulatory element-binding factor
SREBP: Sterol regulatory element-binding protein
TAG: Triacylglycerol
UFA: Unsaturated fatty acid(s)
VFA: Volatile fatty acid(s)
VLDL: Very-low density lipoproteins
VLDLR: Very-low density lipoproteins receptor
